# Multi-tissue Analysis of Co-expression Networks by Higher-Order Generalized Singular Value Decomposition Identifies Functionally Coherent Transcriptional Modules

**DOI:** 10.1371/journal.pgen.1004006

**Published:** 2014-01-02

**Authors:** Xiaolin Xiao, Aida Moreno-Moral, Maxime Rotival, Leonardo Bottolo, Enrico Petretto

**Affiliations:** 1 Medical Research Council (MRC) Clinical Sciences Centre, Faculty of Medicine, Imperial College, London, United Kingdom; 2 Department of Mathematics, Imperial College, London, United Kingdom; Georgia Institute of Technology, United States of America

## Abstract

Recent high-throughput efforts such as ENCODE have generated a large body of genome-scale transcriptional data in multiple conditions (e.g., cell-types and disease states). Leveraging these data is especially important for network-based approaches to human disease, for instance to identify coherent transcriptional modules (subnetworks) that can inform functional disease mechanisms and pathological pathways. Yet, genome-scale network analysis across conditions is significantly hampered by the paucity of robust and computationally-efficient methods. Building on the Higher-Order Generalized Singular Value Decomposition, we introduce a new algorithmic approach for efficient, parameter-free and reproducible identification of network-modules simultaneously across multiple conditions. Our method can accommodate weighted (and unweighted) networks of any size and can similarly use co-expression or raw gene expression input data, without hinging upon the definition and stability of the correlation used to assess gene co-expression. In simulation studies, we demonstrated distinctive advantages of our method over existing methods, which was able to recover accurately both common and condition-specific network-modules without entailing *ad-hoc* input parameters as required by other approaches. We applied our method to genome-scale and multi-tissue transcriptomic datasets from rats (microarray-based) and humans (mRNA-sequencing-based) and identified several common and tissue-specific subnetworks with functional significance, which were not detected by other methods. In humans we recapitulated the crosstalk between cell-cycle progression and cell-extracellular matrix interactions processes in ventricular zones during neocortex expansion and further, we uncovered pathways related to development of later cognitive functions in the cortical plate of the developing brain which were previously unappreciated. Analyses of seven rat tissues identified a multi-tissue subnetwork of co-expressed heat shock protein (Hsp) and cardiomyopathy genes (*Bag3*, *Cryab*, *Kras*, *Emd*, *Plec*), which was significantly replicated using separate failing heart and liver gene expression datasets in humans, thus revealing a conserved functional role for Hsp genes in cardiovascular disease.

## Author Summary

Complex biological interactions and processes can be modelled as networks, for instance metabolic pathways or protein-protein interactions. The growing availability of large high-throughput data in several experimental conditions now permits the full-scale analysis of biological interactions and processes. However, no reliable and computationally efficient methods for simultaneous analysis of multiple large-scale interaction datasets (networks) have been developed to date. To overcome this shortcoming, we have developed a new computational framework that is parameter-free, computationally efficient and highly reliable. We showed how these distinctive properties make it a useful tool for real genomic data exploration and analyses. Indeed, in extensive simulation studies and real-data analyses we have demonstrated that our method outperformed existing approaches in terms of efficiency and, most importantly, reproducibility of the results. Beyond the computational advantages, we illustrated how our method can be effectively applied to leverage the vast stream of genome-scale transcriptional data that has risen exponentially over the last years. In contrast with existing approaches, using our method we were able to identify and replicate multi-tissue gene co-expression networks that were associated with specific functional processes relevant to phenotypic variation and disease in rats and humans.

## Introduction

The increasingly cheaper and rapid accumulation of large -omics datasets across several experimental conditions has prompted generation of a wealth of data on biological networks. This growth of network data now permits their large scale applications to biomedical research, including analysis of gene function, metabolic and signaling pathways, as well as disease-related or cell function-related networks [1], [2]. However, reconstructing and interpreting large biological networks, such as co-expression networks, protein-protein interaction networks or genetic networks, with different features (e.g., sparse or densely interconnected, etc.) poses many challenges, advocating efficient and flexible methods for network inference and pattern discovery. An important level of complexity in current network analysis regards its extension to multiple conditions, for instance different species [3], cell-types [4] or disease states [5], [6]. For example, reconstruction of networks across multiple disease-states is becoming a useful approach for efficient drug-target discovery, as networks can inform the “biological context” (e.g., pathways, cellular processes) where genes operate and therefore can help designing better therapeutic interventions [7]. In genetic studies of complex diseases researchers increasingly focus on groups of highly interconnected genes within larger networks (referred to as clusters, modules or subnetworks) to elucidate specific cellular and molecular processes that might represent functional disease mechanisms and pathological pathways [8]–[10].

While several computational tools for network analysis in single datasets or conditions are available, only few computationally efficient methods for genome-scale network analysis across multiple conditions have been developed to date. These methods can be broadly classified into two main categories: (i) methods to find the “difference” between networks across conditions or to pinpoint condition-specific networks [11]–[14], or (ii) methods to identify the common parts in networks across conditions [15]–[17]. More recently, tensor-based computational frameworks [15] or probabilistic Markov blanket search algorithms [18] have been proposed to learn network structures across conditions. However, these methods are either heavily influenced by the choice of input parameters (e.g., number of clusters, number of nodes within a cluster, cluster interconnectivity) [15] or, being based on probabilistic graphical modelling, they become prohibitively slow for high number of conditions since they are trying to learn the structure of large graphs [18].

Complementary to the above approaches, spectral methods, such as Singular Value Decomposition (SVD), have been also proposed to investigate patterns of connectivity between nodes within a single network [19], [20] or for comparing two networks [21]. Generally, any network can be described as a graph, which is denoted as 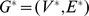 comprising a set 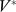 of vertices or nodes together with a set 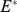 of edges [22]. The graph may be represented by a square, symmetric, real-valued matrix 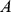 of size 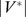 whose entries denote the relationship between the corresponding nodes. In the affinity matrix 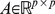, the element 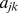, called weight, represents the strength of connection between vertices 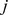 and 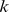. For instance, in gene regulatory (or co-expression) networks, the nodes might represent genes (or mRNAs expression) and edges represent the strength of gene-gene interactions (or mRNAs co-expression).

Generalized Singular Value Decomposition (GSVD) can be used to identify sub-network structures and for comparative analysis of genomic datasets across two conditions [11], [23]. Given two matrices 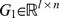 and 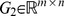 [24], [25], their GSVD is given by
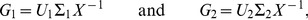
(1)where 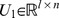 and 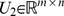 have orthonormal columns, 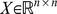 is invertible, 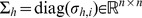 with 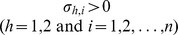, 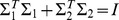 with 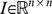. The ratios 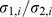 are the *generalized singular values* of 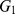 and 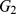. In this setup, the common factor 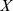 is informative of the cluster structure shared across the two data matrices.

Recently, a novel mathematical formulation, higher-order GSVD (HO GSVD), which is constructed for more than two data matrices has been proposed [26]. Under this framework, the 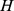 matrices 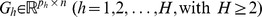, each with full column rank (i.e., the maximum number of linearly independent column vectors of 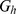 is 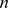), are decomposed as
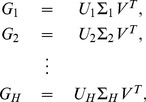
(2)where 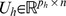 is composed of normalized left basis vectors, 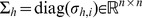 with 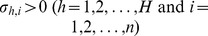 and the latent factor matrix 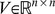 is composed of normalized right basis vectors. The HO GSVD can be also derived in the special case of square, symmetric, full rank affinity matrices, 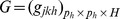, where each element 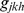 represents the weight of the edge between node 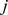 and 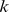 in the 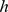
th condition. It has been previously employed to compare multiple datasets with identical column size in order to detect their common substructures of columns (i.e., observations) [26]. Yet, another useful application of the HO GSVD to genomics is to set it to discover gene networks across multiple conditions and pinpoint “common” and “differential” cluster structures.

In this paper, we build on the flexible HO GSVD mathematical framework and propose a new, parameter-free computational algorithm (Cross-Conditions Cluster Detection or C3D) for automatic detection of both similarity and dissimilarity clustering patterns in large weighted (and unweighted) networks across several conditions (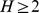). The original HO GSVD model has been employed for analysis of datasets 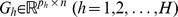 that had varying number of genes (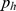), the same number of observations (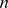) (i.e., arrays/time points in [26]) across conditions and with 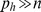. As such, this illustrative application of the HO GSVD in genomics was aimed at the identification of common structures within the 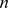 observations [26]. Here, we built on the initial HO GSVD to extract sub-structures (i.e., common and differential clusters) from 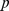 genes across multiple conditions (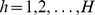) by applying the decomposition to the transposed expression matrix 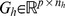. We show how this enables a more general application of the HO GSVD framework to genome-scale network analysis of genomic data (e.g., microarray, RNA-seq) in multiple conditions. Besides, a distinctive feature of our method is in its capability to take as an input either the raw expression matrices or co-expression matrices, allowing flexibility in the choice of the co-expression measures (e.g., Spearman, Kendall, mutual information, etc.).

**Figure 1:**
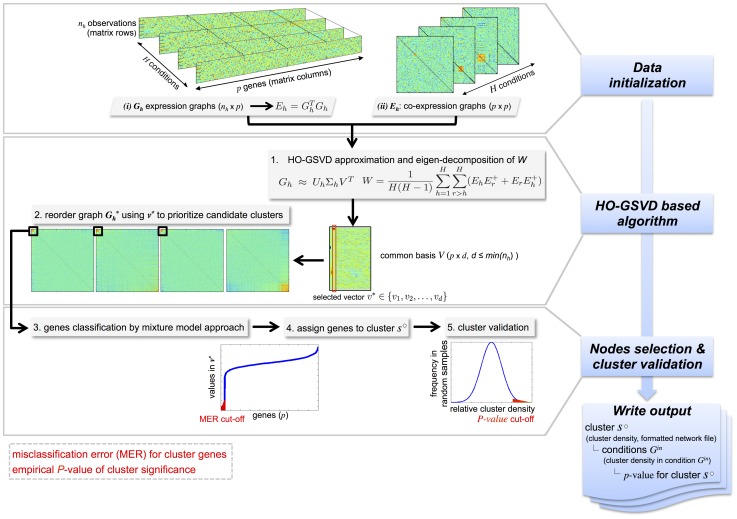
Illustration of the C3D method. Graphical summary of the main steps of the C3D method: (1) data initialization, (2) HO-GSVD based algorithm and (3) cluster nodes selection and validation. Input data can be either gene expression or co-expression matrices (graphs) and the output include information about the identified clusters (cluster density, formatted network file), the conditions where the clusters are detected and the cluster significance (*p*-value). To retrieve significant clusters, the user can specify (i) the misclassification error rate (MER) for inclusion of genes in the cluster and (ii) the empirical *p*-value for significance of the cluster.

Figure 1 illustrates the working principle of the C3D algorithm. The input data for C3D can be provided into different formats to be used by the HO GSVD: (i) the raw expression data matrices (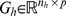) or (ii) the co-expression data matrices (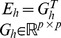). In the former case, a first *data initialization* step is conducted where the input expression matrices, with the same number of genes 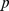 are converted to co-expression matrices 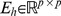 by scaling their variance to 1 and taking their quadratic form. In the second step (*HO GSVD-based algorithm*), an approximate HO GSVD is employed to identify a common basis 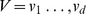, with 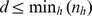 representing the dimension of the GSVD common subspace, for the decomposition of the input datasets and identify the common and differential correlation structures. The HO GSVD-based algorithm computes a 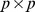 square matrix 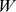, which is built on the arithmetic mean of all pairwise quotients 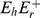 where 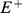 denotes the Moore-Penrose inverse of the co-expression matrix 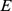 [24] (see *Methods* section). The first eigenvectors of 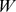 (according to the norm of the corresponding eigenvalues) are then used to identify an approximate decomposition of the input co-expression matrices and form the decomposition basis 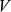. Specifically, each selected column vector of 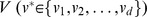 is used to reorder the input data matrices such that candidate “common” (or “differential”) clusters can be identified. In the third step (*cluster nodes selection and validation*), we employ a mixture model approach to classify genes and assign them to each cluster based on a misclassification error rate (MER). Finally, we implemented an empirical cluster validation procedure to identify the conditions where clusters are present and assess the level of significance for clusters within each condition.

To demonstrate the increased power and benefits of our HO GSVD-based algorithm, we carried out an extensive simulation study and benchmarked C3D against commonly used methods that were designed to detect either common (WGCNA [16], [17]) or differential network structures (DiffCoEx [13]) across multiple conditions. We show that our approach has higher power and stability in detecting both common and differential co-expression clusters across all simulated conditions, while being two to seven fold less computationally intensive than alternative methods. In contrast with alternative approaches that require specification of *ad-hoc* input parameters, the proposed method has the distinctive advantage of being parameter-free, which makes it a powerful tool for real data exploration and analysis. To substantiate this claim, we applied C3D to publicly available transcriptomic datasets in rats and humans and identified several multi-tissue gene co-expression networks that were associated with specific functional processes relevant to phenotypic variation and disease.

## Results

### Simulation studies

We carried out a simulation study to compare our method with commonly used approaches for identification of “common” or “differential” clusters across multiple networks: (1) WGCNA and (2) DiffCoEx. The WGCNA method for detection of common clusters across co-expression networks employs a “soft” threshold to assign a connection weight to each gene pair and extract densely connected gene clusters that are present in all conditions. The DiffCoEx method follows a strategy similar to WGCNA but, instead, it focuses on detecting the differences in co-expression patterns (“differential” clusters) between multiple conditions. Additional details on the specific parameterizations used in for WGCNA and DiffCoEx analyzes are reported in Text S1.

**Figure 2:**
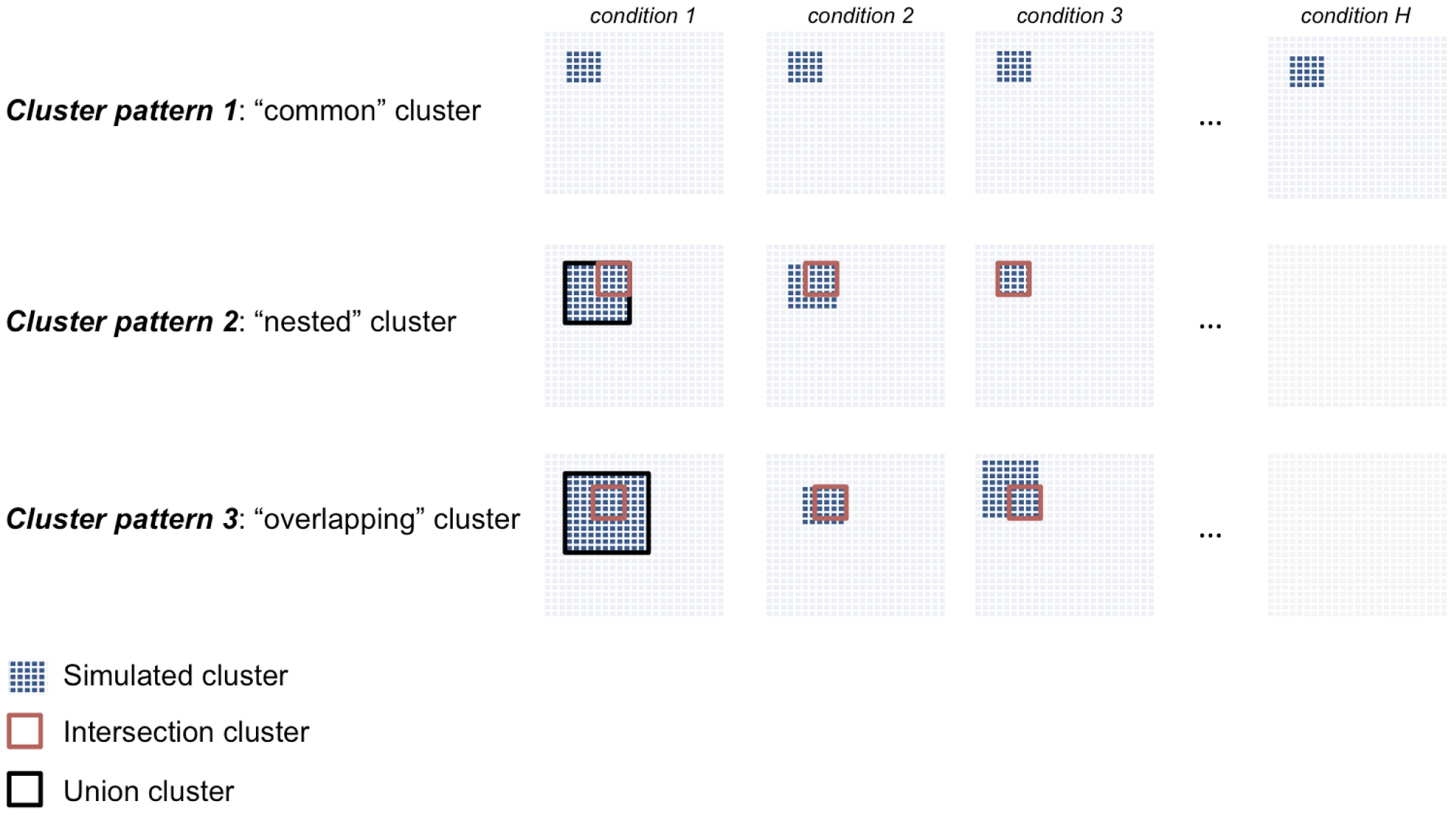
Description of the cluster structures used in the simulation studies. We simulated three cluster types: “common” (*Cluster pattern 1*), “nested” (*Cluster pattern 2*) and “overlapping” (*Cluster pattern 3*) that are shared across three or more conditions. For *Cluster pattern 2* and *Cluster pattern 3*, the “intersection cluster” is defined by the nodes in common to all conditions (red square) whereas the “union cluster” is defined by the nodes in common to all conditions plus the nodes present in individual conditions (black square).

To simulate a realistic example of gene expression data from multiple conditions that represent a typical “small 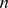 large 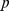
” scenario, we draw inspiration from a publicly available multi-tissue microarray dataset consisting of genome-wide expression profiles from 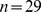 recombinant inbred rat strains in seven tissues [27]. We simulated different types of clusters that are either detected in all conditions (“common” clusters) or are specific to a subset of conditions (“differential” clusters), Figure 2. We considered dense clusters of variable sizes (100–500 nodes) where each node is connected with *all* other nodes in the cluster with a given weight (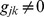), which is defined as the Pearson correlation between expression profiles of genes 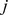 and 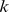. We simulated clusters with varying cluster densities (0.1, 0.3, 0.5, 0.7), which were defined as the average Pearson correlation between any pair of nodes within a cluster. In addition to the simple case of a cluster common to all conditions and with the same size (*Cluster pattern 1*), we set out to evaluate the sensitivity of our and alternative approaches to detect clusters which are present only in a subset of conditions and that overlap partially across conditions. This is more likely to be relevant for analysis of pathways and gene networks across tissues or during development, where varying gene-sets can exert their function only at specific developmental times or in specific cell-types. To account for these more complex scenarios, we simulated “nested” (*Cluster pattern 2*) and partially “overlapping” (*Cluster pattern 3*) cluster structures (Figure 2). *Cluster pattern 2* and *Cluster pattern 3* have an *intersection part*, defined by the nodes in common to all conditions, and a *union part*, defined by the nodes in common to all conditions plus the nodes present in individual conditions. In summary, for each of the four cluster densities considered one dataset consisted of a 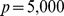 and 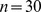 matrix in 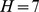 conditions, where each cluster type (*Clusters patterns 1–3*) was simultaneously present in the data matrix. To assess reliability of the results, for each of these data we generated 20 independent replicates, yielding a total of 560 simulated datasets. Similarly, to evaluate how the number of available observations affects the methods' performance we simulated datasets consisting of a 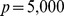 and 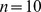 matrix in 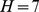 conditions (20 replicates, 560 datasets in total). See Text S1 for additional details.

### Comparison with other methods

The True Positive Rate (TPR) and the False Positive Rate (FPR) are widely used as evaluation metrics for a classification model and can be used to quantitatively assess (and compare) methods performance [28]. The TPR defines how many correct positive results (simulated clusters genes within the called cluster) occur among all results called positive in the analysis by a given method. FPR, on the other hand, defines how many incorrect positive results occur among all results called positives. Typically, a 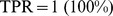 and the corresponding 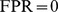 indicate a perfect classifier (or a perfect method). In our simulation study, the best cluster detection method would yield both high TPR and low FPR levels for different cluster types, sizes and densities.

**Figure 3:**
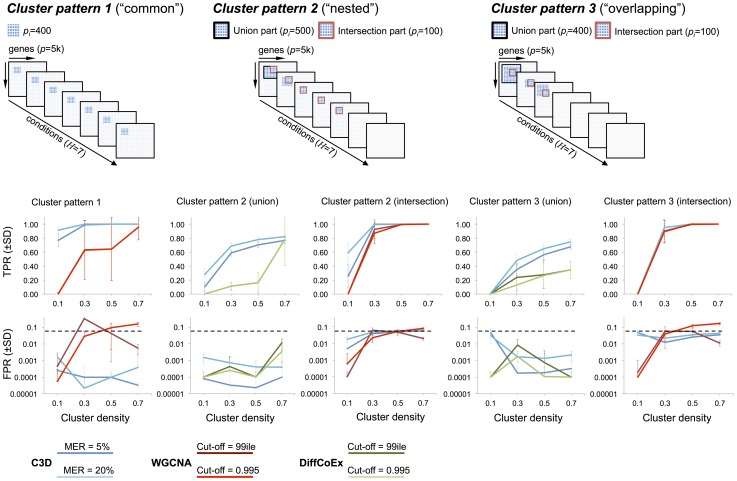
Performance comparison for C3D, WGCNA and DiffCoEx methods *Top*, three cluster types (“common” “nested” and “overlapping”) were simulated in 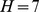 conditions where the cluster size (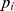) is reported for both the intersection and union part of the clusters. *Bottom*, for each method the average TPR and FPR (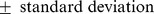) across 20 replicated datasets were calculated and reported for the simulated cluster densities. For C3D analysis (blue lines) we required each cluster to be detected with a misclassification error rate (MER) of 5% or 20% and 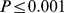. For WGCNA (red line) and DiffCoEx (green line) we considered two “default values” for the cut-off threshold, which were chosen according to the WGCNA guidelines (see Text S1 for details).

For each simulated cluster type, Figure 3 shows the TP/FP rates for C3D, WGCNA and DiffCoEx methods as a function of the simulated cluster densities. For C3D we controlled the (local) misclassification error (i.e., the probability to assign wrongly a gene to a cluster) to be less than 0.05 or less than 0.2, and required that each cluster is detected with 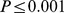, whereas for WGCNA and DiffCoEx we used two (default) parameterizations chosen according to the software guidelines (see *Methods* section). The C3D method outperformed WGCNA in the identification of clusters present in all conditions (*Cluster pattern 1*, Figure 3), and showed to have consistently high TPR (and very low FPR, 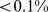) irrespective of the simulated cluster density. WGCNA performance varied considerably as a function of the simulated cluster density and, depending on the adopted parameterization, FPR levels were 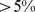 (reaching 20% in one case), Figure 3. Furthermore, we observed large variations in WGCNA performance (mostly in the TPR), which are indicated by the large standard deviations in TPRs calculated from the 20 replicated datasets. For more complicated patterns (“nested” and “overlapping” clusters), we compared C3D with WGCNA to detect the *intersection part* (100 nodes) of common clusters. Since WGCNA is designed to detect only those clusters shared across all conditions, for clusters present in a subset of conditions, we run WGCNA only in the set of conditions where the simulated clusters were present. For *Cluster patterns 2–3*, C3D and WGCNA performances were similar, reaching high TPR for detection of the intersection part of clusters with simulated 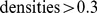 (Figure 3). However, C3D showed higher TPRs than WGCNA to detect clusters with low densities (0.1–0.3), while controlling the FPR at low levels (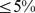, *Cluster pattern 2* intersection).

In the case of partially overlapping clusters present in a subset of conditions (*Cluster patterns 2–3*) we compared C3D with DiffCoEx in respect of detecting the *union part* (500 nodes) of “differential” clusters, and calculated TPR and FPR for detection of this cluster (indicated with a black square at the top of Figure 3). We found that C3D outperformed DiffCoEx across the simulated scenarios. In the case of the “nested” cluster structures that are present in 5 out of 7 conditions, C3D had consistently higher TPR levels than DiffCoEx, which showed comparable TPR levels only for detection of highly-dense clusters (i.e., 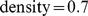, *Cluster pattern 2* union, Figure 3). However, similarly to what observed for WGCNA method, in this case DiffCoEx showed large variability in its performance across the 20 replicated datasets. The difference in performance between C3D and DiffCoEx was observed also in the more complicated case of partially overlapping cluster structures (*Cluster pattern 3*). In this case, C3D showed consistently higher TPR than DiffCoEx that reached a maximum 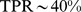 as compared with 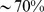 of C3D. Both methods showed comparably low FPR (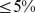) for detection of the union part of *Cluster patterns 2–3* (Figure 3). Similarly to what observed for the simulated data with 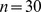 observations, C3D performed better than (or as good as) both WGCNA and DiffCoEx when benchmarked on simulated datasets with only 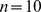 observations (Figure S1). As expected, all methods had lower TPRs associated with the detection of low-density clusters, however also with a small number of observations, C3D showed significantly better (and more stable) results than WGCNA and similar performance as compared with DiffCoEx. Notably, for detection of “common” clusters present in all conditions (*Cluster pattern 1*), CD3 held high TPR levels (and 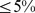 FPR) whereas WGCNA's performance dropped significantly, reaching a maximum 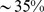 TPR (Figure S1).

These data show that C3D on balance performed better than WGCNA and DiffCoEx across all simulated scenarios. We underline that while WGCNA and DiffCoEx methods are specifically designed to detect either common or differential clusters, respectively, here we showed that C3D was equally or more accurate than both methods in the detection of common *and* differential cluster structures. We also highlight how C3D ability to detect correctly the simulated clusters was highly consistent across all runs on the replicated datasets, as shown by the small standard deviations of the mean TP and FP estimates (Figure 3). In contrast, we observed that both WGCNA and DiffCoEx performances varied appreciably across the replicated simulations, often resulting in large standard deviations of the mean TP and FP estimates. To better assess the reliability of the different methods we calculated the relative standard deviation 
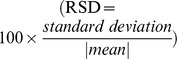
 of the TPR measured in all analyzed datasets. In 560 simulated datasets of size 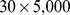, the C3D method had a median RSD of 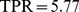 (range 113.36) whereas WGCNA and DiffCoEx have median 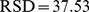 (range 447.2) and median 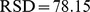 (range 133.39), respectively. Similarly, in 560 datasets of size 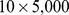 we estimated the following RSDs of TPR: 12.43 (range 113.38) for C3D, 57.52 (range 161.89) for WGCNA and 87.96 (range 120.59) for DiffCoEx. The large RSDs of TPR calculated from the WGCNA and DiffCoEx analyzes originated because these methods often detected the simulated cluster(s) only in small number of replicates (e.g., 2 out of 20).

Besides, in a few cases the TP/FP rates of WGCNA and DiffCoEx were influenced by the adopted parameterization (for instance, FPR in the WGCNA analysis of *Cluster pattern 1*, Figure 3), suggesting that different choices of the input parameters can affect the detection of clusters (see Text S1 for additional details). The C3D algorithm is built on the HO-GSVD framework and as such does not require the user to specify *ad-hoc* parameters to detect common or differential clusters. In our implementation of the C3D algorithm the user can control the MER at a specified level before the cluster genes are empirically validated using a permutation-based procedure (see *Methods* section). In these simulation studies, we have used two different MERs (5% and 20%) to inform a suitable choice of MER that maximizes true positive without inflating false positive rates. On average, we observed a 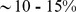 increase in the TPR when 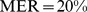 was adopted as compared with 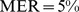. However, we found no significantly higher FPR, which were always 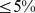 across all simulated datasets, this suggesting that using the less stringent 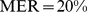 in real data analyzes is likely to increase the detection of true gene clusters, without increasing significantly false positives.

Finally, we used a standard desktop computer (Mac Pro, 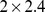 GHz Quad-core Intel Xeon with 20 Gb RAM) to evaluate the computational time required by C3D and compare it with WGCNA and DiffCoEx to analyze the simulated datasets. While the run time of C3D scales exponentially with the number of genes in the input matrices or the number of conditions, our Matlab implementation of C3D is relatively fast and requires only 1,200s to analyze a 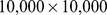 gene co-expression matrix in 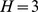 conditions and 10s to analyze a 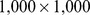 gene co-expression matrix in 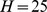 conditions (Figure S2). When compared with competing approaches, we assessed that to process simulated datasets of 1,000 and 10,000 genes (with 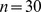 observations and 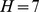 conditions) C3D requires significantly smaller CPU time than DiffCoEx (up to 2.3 fold more CPU time) and WGCNA (up to 8.2 fold more CPU time), respectively (Figure S2).

### Case studies

To show how C3D provides a powerful, practical framework for real genome-scale analyzes and yields new biological insights into pathways and molecular networks, we report an application to two large multi-tissue gene expression datasets in rats and humans. Transcriptional profiling was carried out by Affymetrix microarray in the rat and mRNA sequencing (RNA-seq) in humans, respectively. The microarray dataset consisted of genome-wide expression profiles (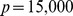 probe sets) that were measured in seven tissues (adrenal, aorta, fat, kidney, left ventricle, liver and skeletal muscle) in a panel of 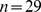 recombinant inbred rat strains [29], which is a well characterized model of hypertension, metabolic syndrome and cardiovascular disease [27], [30], [31]. The RNA-seq datasets consisted of genome-wide transcriptomic data of human fetal neocortex, which have been generated to investigate the molecular mechanisms underlying differences in germinal zones of the developing human brain. The human dataset consisted of 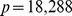 expressed genes which were analyzed in four regions of the fetal neocortex (ventricular zone (VZ), inner subventricular zone (ISVZ), outer subventricular zone (OSVZ) and cortical plate (CP)) from six 13–16 weeks postconception human fetuses [32]. In both rat and human analyzes, to identify common and differential clusters we extracted the top ten eigenvectors (based on the modulus of the eigenvalues of the decomposition of 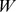) as candidates which are then used as input for the *cluster nodes selection and validation* step of the C3D algorithm (see Methods).

#### Transcriptional network analysis in seven rat tissues

We employed a two-step strategy to identify co-expression clusters present in all (or in a subset of) tissues: (i) we prioritize candidate gene clusters using a “relaxed” 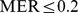 to assign genes to each cluster (see *Methods* section) and then (ii) used the permutation-based procedure (integrated in C3D) to select significant clusters and identify the relevant tissues using a stringent empirical *P*-value threshold (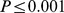). This strategy yielded a set of 8 gene co-expression clusters: 3 clusters were detected in all tissues and 5 clusters were specific to a sub-set of tissues (Table S1). We set out to systematically analyze these gene co-expression clusters using four approaches: (i) functional enrichment analysis using Gene Ontology and KEGG pathways [33], (ii) cell-type specificity using Cell Type ENrichment (Cten) analysis for microarray data [34], (iii) cluster conservation with experimentally validated protein-protein interactions (PPI) and protein complexes using the DAPPLE algorithm [35] and (iv) enrichment of transcription factor binding sites (TFBSs) in the putative promoter sequences of cluster genes using the Pastaa algorithm [36]. (See Text S1 for additional details on cluster annotation and analysis).

**Figure 4:**
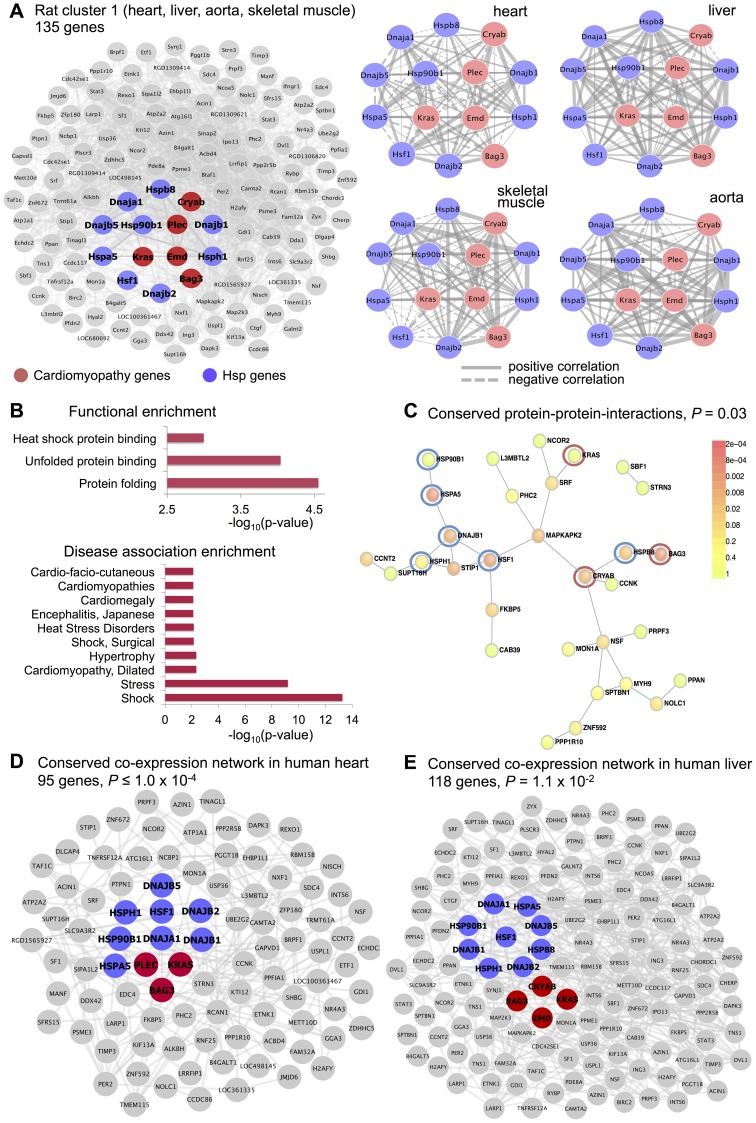
*Rat cluster 1* shows co-expression between Hsp and cardiomyopathy genes which is conserved with human heart and liver tissues (A) Network of 135 annotated rat genes identified by C3D as co-expressed in heart, aorta, liver and skeletal muscle tissues (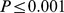). In each tissue we selected the top 5% of edges based on the (absolute) covariance between gene expression profiles and then calculated the average covariance across the four tissues. Edges are represented by lines connecting nodes (genes) and the thickness of the line is proportional to the average covariance value. Within the network, heat shock protein (Hsp) and cardiomyopathy genes are highlighted in blue and red, respectively. The Kendall correlations between the expression profiles of Hsp and cardiomyopathy genes are graphically represented as sub-networks separately for each tissue. Line thickness is proportional to the value of the Kendall correlation. (B) Enrichment for functional categories (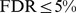, full list in Table S2) and for disease association (adjusted 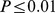, details in Table S3). (C) Significant protein-protein interaction (PPI) network (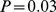) where the Hsp and cardiomyopathy genes showing conserved PPI are highlighted (blue and red circles). (D) Conserved co-expression network detected in 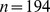 heart tissue samples from patients with advanced idiopathic or ischemic cardiomyopathy. The network includes all human orthologous genes of the genes in *rat cluster 1* that have significant edges by covariance selection (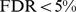). (E) Conserved co-expression network detected in 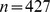 liver tissue samples from healthy volunteers. The network includes all human orthologous genes of the genes in *rat cluster 1* that have significant edges by covariance selection (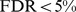).

One large “differential” cluster consisting of 172 microarray probe sets (*rat cluster 1*) was identified in skeletal muscle, left ventricle, aorta and liver tissues (empirical 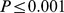, Figure 4A). This cluster showed significant enrichment for “protein folding” (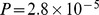), “unfolded protein binding” (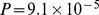) and “heat shock protein binding” biological processes (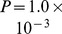), Figure 4B, but did not revealed strong enrichment for either specific cell-types or TFBSs in the cluster genes promoter (Table S2). We found that *rat cluster 1* included several heat shock protein (Hsp) genes (*Hsp90b1, DnaJ (Hsp40) homologs, Hspa5, Hspb8, Hsph1*) and the *Hsf1* (heat shock transcription factor 1), which binds to the heat shock element in the promoters of Hsp genes and induce their activation [37]. Heat shock transcription factor 1 is a crucial transcription factor for heat shock proteins and appears to serve a significant protective role in the heart [38], [39]. Besides, closer inspection of *rat cluster 1* reveal genes known to have disease mutations in hereditary cardiomyopathy in humans (*Bag3, Cryab, Kras, Emd, Plec*) [40] (Figure 4A). Therefore, we investigated whether *rat cluster 1* genes have been previously implicated in disease using the gene set analysis toolkit WebGestalt [41], which relies on existing biomedical literature to retrieve accurate disease-associated gene lists [42]. This analysis revealed marked and specific enrichment for genes associated with circulatory shock, stress and cardiac conditions (e.g., cardiomyopathies, hypertrophy, cardiomegaly), Figure 4B and Table S3. Our C3D analysis suggests that cardiomyopathy genes are co-expressed with Hsp genes across several rat tissues including tissues enriched for myocytes (skeletal muscle, heart and aorta) and in the liver, where Hsp genes are known to be expressed in response to a variety of stressful stimuli [43] or to an increase in body temperature [44]. Moreover, several mRNA-mRNA interactions between Hsp and cardiomyopathy genes of *rat cluster 1* were conserved at the protein level (Figure 4C). We then investigated whether *rat cluster 1* genes were significantly conserved and co-expressed in human heart and liver tissues. To this aim, we carried out genome-wide co-expression network analysis using covariance selection models [45] in two large, publicly available gene expression datasets in the heart (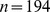 patients with advanced idiopathic or ischemic cardiomyopathy, GSE5406 from Gene Expression Omnibus (GEO) [46]) and liver tissue (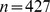 healthy subjects, GSE9588 from GEO [47]). After computing the matrix of partial correlations between the genes' expression profiles in each tissue separately, we tested whether the human-rat orthologous genes of *rat cluster 1* had significant connections (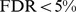) in heart and liver tissues more than what expected by chance. Sampling 10,000 random networks from each partial correlation matrix we found that 95 and 108 human-rat orthologous genes have significantly high interconnectivity in heart (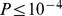) and liver (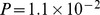) tissues, respectively (Figure 4D and 4E, and Figure S3). This analysis provides independent replication of *rat cluster 1* in two separate datasets and confirms significant co-expression between Hsp and cardiomyopathy genes in human heart and liver tissues. Elevated Hsp gene expression was previously observed in the heart of patients with dilated cardiomyopathy [48], [49] and our data showing conserved co-expression between Hsp and cardiomyopathy genes in rats and humans suggest a potential role for heat shock proteins in cardiovascular disease [50], [51].

**Figure 5:**
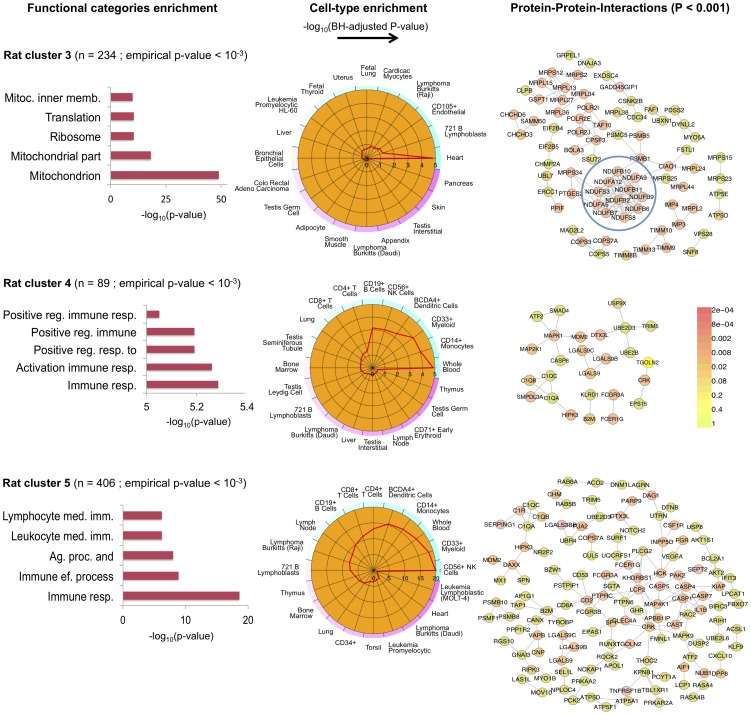
Co-expression clusters identified in all rat tissues. For each rat cluster detected in all seven tissues we report the number of probe sets, the top five functional categories and their statistical significance (full list in Table S2), the summary of cell-type enrichment statistics expressed as 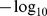 (Benjamini and Hochberg (BH)-adjusted *p*-value, Cten analysis) and the graph with the significant protein-protein interactions (PPI), including the overall significance of the directed PPI network (DAPPLE analysis). The colour scale on the right indicate the significance of the detected PPI.

We identified three co-expression gene clusters consisting of 234, 89 and 406 microarray probe sets, which were detected in all tissues (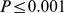, Figure 5 and Table S1). In contrast with the tissues-specific clusters, all multi-tissue clusters were highly conserved at the protein level where they show significantly high protein-protein interconnectivity by DAPPLE analysis (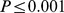, Figure 5). These clusters might represent shared gene-gene interactions and gene expression signatures of fundamental molecular processes, which are strongly conserved at the protein level. These shared gene expression signatures are less likely to be detected in individual tissues where local regulatory mechanisms (translational and post-translational) are likely to be more important [52], [53]. One of these multi-tissue clusters (*rat cluster 3*) included 234 probe sets (representing 214 annotated protein coding genes) and showed a striking enrichment for mitochondrial related genes (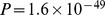), enrichment for heart (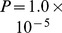) and lymphoblasts (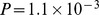) cell-types (Figure 5). This cluster was also significantly overrepresented for the “oxidative phosphorylation” KEGG pathway (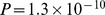), which is an integrative function of mitochondria and that in muscle and heart in controlled essentially at the level of the respiratory chain [54]. At the protein level, we found that *rat cluster 3* identified two important protein complexes: the mitochondrial NADH-Ubiquinone Oxidoreductase (Complex I) (blue circle, Figure 5) and several mitochondrial ribosomal, large subunits, which is consistent with the observed functional/cell-type annotation of the co-expressed gene cluster. Lastly, we identified two common clusters (*rat cluster 4*, *rat cluster 5*) that were most highly enriched for immune response genes and specifically expressed in whole blood and myeloid cell-types (Figure 5). In particular *rat cluster 5* recapitulates a previously identified co-expression network detected in seven tissues (*Irf7*-driven inflammatory gene network or IDIN) [27], which comprised 209 genes directly (and indirectly) regulated by the Irf7 transcription factor (a master regulator of the type 1 interferon response [55]). The multi-tissue cluster identified by C3D was most highly enriched for genes related to “immune response” (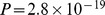) and expressed in myeloid and blood cell-types (*P*-value range from 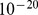 to 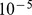). This co-expression network, which is highly expressed in immune cells, may represent a molecular signature of macrophages in complex tissues and is associated with risk of inflammatory diseases and autoimmune disease Type 1 diabetes in humans [56], [57], as previously demonstrated [27]. *Rat cluster 5* was also highly enriched for known protein-protein interactions (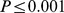), and cluster genes promoters contained TFBS motifs for the IRF transcription factor family (TFBS enrichment 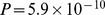, Table S2). We highlight that this inflammatory network (IDIN) was previously identified by complex integration of genome-wide TFBS predictions, expression QTL mapping using genome-wide SNPs and co-expression network analysis in seven rat tissues, and was experimentally validated and translated to humans [27]. Here, we uncovered most of the IDIN (136 genes, 65%) and revealed many key properties of this transcriptional network (functional enrichment, cell-type specificity, IRF-dependent regulation) using only the C3D approach on the gene expression data from seven tissues.

#### Transcriptional network analysis in human brain regions

We set out to identify co-expression gene clusters across human fetal neocortical regions: VZ, ISVZ, OSVZ and CP (RNA-seq datasets: 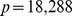 genes in 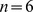 fetuses across 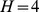 regions). Similarly to the analysis of the rat microarray data, we have used a two-step strategy to first prioritize candidate clusters (using 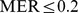) and then validate the clusters by permutations and pinpoint the neocortical regions where these clusters are present (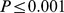). The clusters were annotated in detail and compared with the large catalogue of differentially expressed genes between fetal cortical zones previously reported in [32].

**Figure 6:**
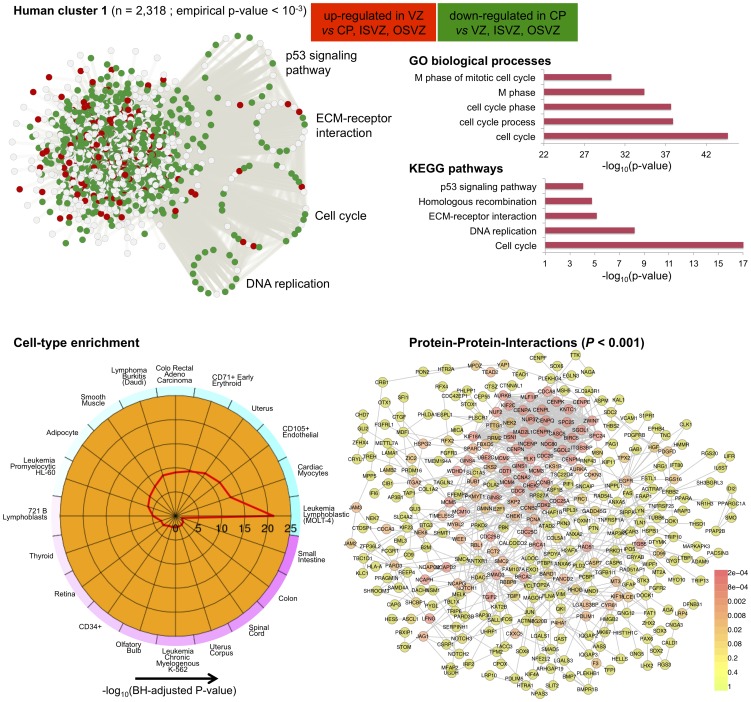
Human co-expression cluster 1. *Top left*, each node in the network represents a gene and, in keeping with [61], for each gene we highlight significant up-regulation in VZ (red) or CP (green) as compared with the other neocortex regions. Genes that are were not differentially expressed between neocortex regions are coloured in grey. Genes present in relevant KEGG pathways (p53 signaling, ECM-receptor interaction, Cell cycle and DNA replication) are extracted from the main network and highlighted. *Top right*, functional annotation for the network: top five significant GO biological processes and KEGG pathways (full list in Table S3). *Bottom left*, summary of cell-type enrichment analysis expressed as 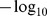 (Benjamini and Hochberg (BH)-adjusted *p*-value, Cten analysis). *Bottom right*, graph with the significant protein-protein interactions (PPI), including the overall significance of the directed PPI network (DAPPLE analysis, 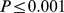). The colour scale on the right indicate the significance of the detected PPI.

**Figure 7:**
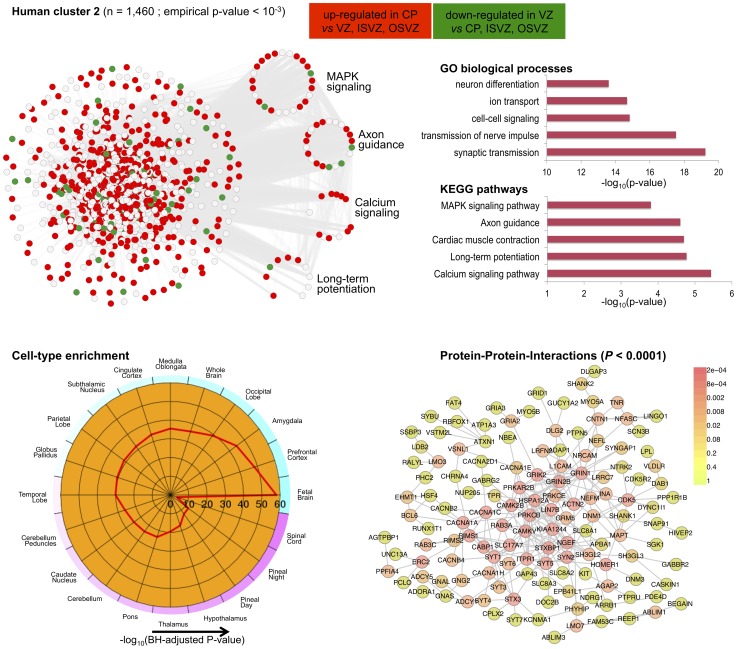
Human co-expression cluster 2. *Top left*, each node in the network represents a gene and, in keeping with [61], for each gene we highlight significant up-regulation in CP (red) or VZ (green) as compared with the other neocortex regions. Genes that were not differentially expressed between neocortex regions are coloured in grey. Genes present in KEGG pathways related to cognitive functions (MAPK signaling, axon guidance, calcium guidance and long-term potentiation) are extracted from the main network and highlighted. *Top right*, functional annotation for the network: top five significant GO biological processes and KEGG pathways (full list in Table S3). *Bottom left*, summary of cell-type enrichment analysis expressed as 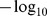 (Benjamini and Hochberg (BH)-adjusted *p*-value, Cten analysis) showing the most significant enrichment for fetal brain, prefontal cortex and amygdala tissues. *Bottom right*, graph with the significant protein-protein interactions (PPI), including the overall significance of the directed PPI network (DAPPLE analysis, 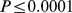). The colour scale on the right indicate the significance of the detected PPI.

The C3D analysis revealed two large clusters (*human cluster 1*, *human cluster 2*) including 2,318 and 1,460 genes, respectively, which were highly enriched (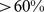 of genes) for differentially expressed genes between the CP and VZ, ISVZ, OSVZ neocortex regions (Table S4). These clusters were identified as “differential” clusters, and were specifically expressed in VZ, ISVZ, OSVZ (*human cluster 1*) and in CP (*human cluster 2*) fetal neocortex regions with a high significance level (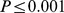). The identification of “differential” clusters between different neocortex regions during development matched the enrichment for differential expressed genes within these clusters, where *human cluster 1* was most highly enriched (1,450 out of 2,318 genes, 63%, hypergeometric enrichment test 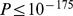) for genes down-regulated in CP as compared with VZ, ISVZ, OSVZ, whereas *human cluster 2* was most highly enriched (940 out of 1,460 genes, 64%, hypergeometric enrichment test 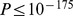) for genes up-regulated in the CP region as compared with VZ, ISVZ, OSVZ (Figure 6 and Figure 7). Gene Ontology annotation of the cluster genes revealed functionally coherent processes with the most significant enrichment for “cell cycle” (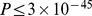) in *human cluster 1* and “synaptic transmission” (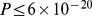) in *human cluster 2*, respectively (Table S5). In particular, *human cluster 1* recapitulates the cell-to-extracellular matrix interactions processes which were previously found to be associated with up-regulation in either VZ, ISVZ or OSVA neocortex regions [32]. However, our multi-tissue network analysis and annotation of the results suggest further functional specialisation of the two clusters which was previously unappreciated.

In particular for *human cluster 1* we found strong co-expression between 1,450 of the differentially expressed genes which are enriched for cell adhesion and cell-extracellular matrix (ECM) interaction processes during cortical development [32]. This co-expression pattern suggests crosstalk between different pathways across neocortex regions, as it is shown here for “cell cycle” and “ECM-receptor interaction” (Figure 6). This is in keeping with the notion that cell cycle progression in mammalian cells is strictly regulated by both integrin-mediated adhesion to the extracellular matrix and by binding of growth factors to their receptors [58]. Surprisingly, cell-type enrichment analysis suggested highly specific expression of *human cluster 1* in MOLT-4 (human T lymphoblast; acute lymphoblastic leukemia) cell line, which constitutively does not express p53 (a key regulator of the cell cycle, DNA repair and cell death). However, since we found down-regulation of p53 signalling and other related pathways, the observed enrichment for MOLT-4 cell-type most likely reflected cell-type-specific depletion of p53 expression and of many target genes in the CP region. Analysis of TFBS motifs in the promoter of *human cluster 1* genes revealed the E2F1 transcription factor (TFBS enrichment 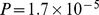), which plays a crucial role in the control of cell cycle regulation/progression and have been implicated in neural stem cell maintenance and commitment [59]. Taken together, these analyzes of *human cluster 1* suggest that differentially expressed genes related to cell-ECM interaction exert their function in a highly coordinated fashion where multiple pathways are involved in cell proliferation and self-renewal of neural progenitors in developing human neocortex.

Similarly to the first cluster, *human cluster 2* was significantly enriched for differentially expressed genes between CP and VZ, ISVZ, OSVZ regions, but in this case with marked up-regulation of gene expression in the CP region (Figure 7). Functional enrichment analysis suggested up-regulation of several KEGG pathways, such as “calcium signaling pathway” and “long-term potentiation” (Figure 7) that are associated with key cognitive functions, including memory and learning. Cell-type enrichment and protein-protein interaction analyzes for *human cluster 2* showed high specificity of this cluster in fetal brain, prefontal cortex, amygdala tissues (enrichment 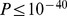), and strong conservation of the network at the protein level (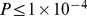), Figure 7. Analysis of TFBS enrichment in the promoter of cluster genes revealed different sets of TFs including neuronal-specific factors like Rest that regulates repression of multiple neuron-specific genes (TFBS enrichment 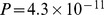) or TFAP2A that is essential for development of sympathetic neurons by controlling the survival of a subpopulation of migrating neural crest cells [60](TFBS enrichment 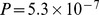), and other myogenic regulatory factors (Myf, TFBS enrichment 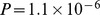) or factors regulating transcriptional events during hemopoietic development (MZF1, TFBS enrichment 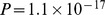). The original investigation of gene expression variation across human fetal neocortexes regions reported in [32] suggested a role for extracellular matrix in progenitor neuronal cells self-renewal. Here, our C3D analysis was able to recapitulate these biological processes and furthermore highlight extensive co-expression between cell-cycle and ECM-interaction genes in proliferation and renewal of neuronal progenitors in specific neocortex regions (*human cluster 1*). In addition, our analysis revealed a distinct functionally-coherent network (*human cluster 2*) related to development of later cognitive functions in developing brain, which was not reported in the original study [32]. These new findings are consistent with recent data on human-specific gene expression changes taking place during postnatal brain development in the prefrontal cortex [61].

## Discussion

Building on the HO GSVD framework, we have developed a new algorithm (C3D) for efficient, parameter-free and automatic detection of co-expression clusters and networks in multiple conditions. Our method is designed for analysis of weighted (and unweighted) networks (input matrices) 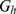 across 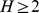 conditions, enabling applications to diverse data types and structures. Although the original HO GSVD algorithm assumes the non-singularity of the co-expression matrix 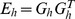, by using the Moore-Penrose pseudo-inverse, our C3D algorithm can be applied to the non-invertible case. We show that when an exact HO-GSVD of the input matrices exists (as defined in (4), see *Methods*), our HO GSVD is able to extract the right decomposition basis 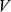 through the eigen-decomposition of 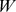, whereas it finds an approximate decomposition of the data in the absence of an exact solution (Figure S4). In particular, our empirical simulations and real-case applications reveal that our approximate decomposition is able to capture both common and differential co-expression structures for a wide range of noise levels, suggesting that our algorithm can be useful for practical applications to genomic data.

Here, through the HO GSVD of large-scale genomic datasets we aimed to uncover the complex interactions between genes (networks) that can occur within or across multiple conditions. One distinctive feature of our computational method is in the flexible and simultaneous identification of both “common” and “differential” sub-network structures across several conditions. Selecting informative vectors of 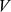, we provide different orderings of 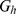 to reveal candidate clusters that are important to all conditions or specific to a sub-set of conditions; then, we can distinguish the specific conditions where the clusters are present using a permutation-based approach. This procedure allows to pinpoint automatically the specific conditions where the sub-network structures are present and, at the same time, to provide an empirical estimate of the statistical significance (empirical *P*-value) for each cluster identified.

In simulation studies, we demonstrated how C3D outperforms competing approaches in accuracy and reliability while being computationally less demanding. We highlight how our method allowed accurate detection of clusters within complex structures (i.e., “common”, “nested” and “overlapping” networks) by specifying only the desired level of statistical significance: misclassification error rate to assign genes to clusters and empirical *P*-value for cluster detection. In contrast with other approaches, C3D does not need the user to specify *ad-hoc* parameters related to the expected number of clusters or cluster density [15] or necessary to determine the optimal height cut-off in the gene clustering tree [13], [16], [17]. Typically, these unknown parameters need to be “finely tuned” on each dataset in order to obtain the best compromise between TP and FP for each cluster (see Text S1 for additional details). We also showed that the results obtained by two competing and widely-used methods (WGCNA and DiffCoEx) were less stable than those provided by C3D. This was apparent in the significantly smaller relative standard deviations in TPR calculated across 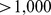 simulated datasets in the C3D analyzes as compared with WGCNA and DiffCoEx. Since C3D utilised raw gene expression data matrices as input, the higher stability of C3D might be due to the reduced influence of the small number of observations on the stability of co-expression estimates, which can result in extreme patterns of correlation changes, corresponding to stable and fragile co-expression, as previously shown [62].

The high stability in the results and the parameter-free “nature” of the HO GSVD approach make the C3D algorithm a powerful computational tool for real genomic data exploration and analysis. To demonstrate this point, we reported an application of C3D to two large transcriptional datasets: (i) microarray-based gene expression profiles in seven rat tissues and (ii) RNA-seq-based gene expression analysis of germinal zones from human fetal neocortex. In the rat analysis, we reported several functionally enriched co-expression clusters, including a previously identified inflammatory gene network driven by the IRF7 transcription factor that represents a gene expression signature of macrophages within complex tissues. While this co-expression network was experimentally validated [27] it was not recovered by WGCNA, that surprisingly placed the IRF7 transcription factor and many regulated target genes in the group of “non-clustered” genes. In addition, our C3D analyzes revealed novel gene co-expression networks in sub-sets of tissues. For instance, we identified a network comprising Hsp and known cardiomyopathy genes, which suggested coordinated regulation of heat shock proteins genes in multiple tissues, and their potential functional role in cardiovascular disease [50]. While this network was not recovered by either WGCNA or DiffCoEx analyzes, we were able to replicate this new finding using separate cardiac and liver gene expression datasets in humans (Figure 4). In the study of human fetal neocortex we demonstrated previously undescribed co-expression between cell cycle and ECM-receptor interaction pathways and support their role in the proliferation and self-renewal of neural progenitors. In addition, our analyzes highlighted that pathways central to later cognitive functions (e.g., calcium signaling, long-term potentiation, axon guidance) are present at an early stage in the developing human brain [61], which was not previously appreciated. These studies illustrated how our method can be effectively applied to leverage the vast stream of genome-scale transcriptional data that has risen exponentially over the last years, promising to aid the fine-scale characterization of both context-specific and systems-level networks and pathways.

## Methods

We describe a new computational method (Cross-Conditions Cluster Detection or C3D) to detect both similarity and dissimilarity clustering patterns in weighted networks across multiple conditions (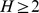). After a *data initialization* step, C3D employs *HO GSVD-based algorithm* and *cluster nodes selection and validation* procedures to identify clusters, the specific conditions where the clusters are detected and the statistical significance of the clusters, as summarized in Figure 1 and detailed below.

### Data initialization

In this step we assume the input data are non-square matrices 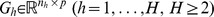, where the 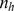 rows represent the observations and the 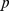 columns indicate genes. The number of genes must be the same across datasets while the number of observations can differ. We first log transform the data and subtract for each gene its average gene expression to avoid capturing differences in average gene expression across conditions. We then calculate the co-expression matrices corresponding to each condition 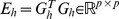. Each 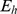 represents the covariance matrix of the data in condition 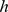. As in classic principal component analysis, the columns of 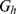 can be scaled to unit variance to work on the correlation matrices rather than the covariance. Alternatively, our algorithm can directly take any 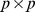 co-expression matrix 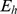 as input. This feature of our algorithm allows to extract common and differential clusters from matrices based on different co-expression measures, including robust correlation (e.g. Spearman, Kendall) and non linear metrics such as mutual information [63].

### The HO GSVD-based algorithm

Similarly to classic SVD, each observation from the input data 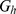 can be characterized by its expression profile and represented by a data point in a 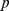 dimensional space. The observations from all datasets are contained in a subspace of dimension 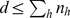, which thereafter is referred to as the HO GSVD subspace. Here, we aim at finding directions in the HO GSVD subspace that either capture the variability in gene expression that is common to all conditions (common factors) or that is specific to a subset of conditions (differential factors). Inspired by [26] we developed a general algorithm that allows computation of an approximate solution to the HO GSVD problem in the non full column rank case. In the HO GSVD, 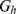 are decomposed into 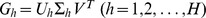 where 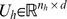, 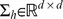 is a diagonal matrix with elements 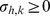 for 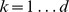 and 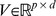 contain the right basis vectors of the HO GSVD subspace where 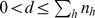. The right basis vectors 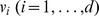 allow to identify set of genes (clusters) with similar co-expression patterns, that are either specific to a subset of conditions or common to all conditions. Here we explain the derivation of our HO GSVD-based algorithm in the general case of 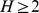 non-square matrices. The derivation and discussion of the special cases (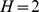 square, symmetric matrices with full rank and 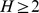 square, symmetric matrices with full rank) is reported in Text S1. In the most general case, we define the right basis vectors 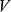 as the solution of the eigen-decomposition problem of the matrix
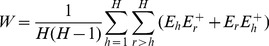
(3)where 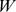 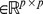 is the arithmetic mean of all the pairwise quotients 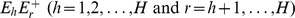 and 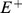 denotes the Moore-Penrose inverse of the co-expression matrix 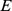 [24]. Here the Moore-Penrose inverse is used as a substitute of 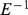 since the invertibility of 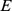 is not guaranteed when 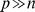, which is the typical scenario in genomics. We now assume there is an approximate HO GSVD 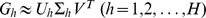 where 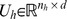 is composed of orthonormal left basis vectors and 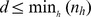. In this case, for all 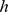 we have
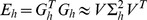
(4)and its Moore-Penrose inverse is given by
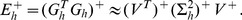
(5)Therefore 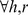 we have
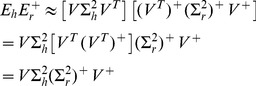
(6)since 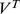 is full row rank. Hence we can rewrite 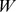 as follows
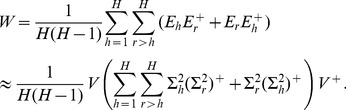
(7)When there exists a common subspace of dimension 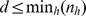, with basis vectors 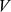, for which the decomposition of the co-expression matrices 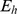 (4) is exact, equation (7) becomes an equality and the eigenvectors of 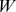 will lead to the exact basis 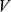 of the common subspace. In HO GSVD applications to genomics data, 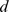 can be as large as the total number of observations (i.e., 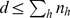), and an exact common decomposition of the co-expression matrices 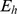 might not be possible. In this case the eigenvectors of 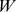 do not provide an exact decomposition of the subspace. Moreover, 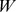 is not guaranteed to be non-defective and have a full set of real eigenvalues and eigenvectors. However, even in the absence of an exact common decomposition, the real part of the complex eigenvectors can be used to derive a low rank approximation of the common subspace and extract common and differential covariance structures from the data. To test the ability of our HO GSVD based algorithm to capture these covariance structures in the data in the presence of a “noisy” HO GSVD decomposition we performed an empirical simulation study (see Text S1 for details). Our simulations suggest that if a common subspace of dimension 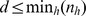 with basis vectors 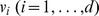 explains a significant fraction of the variance in the original datasets 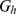, the approximation (4) holds and the first eigenvectors of the matrix 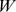 (corresponding to the largest eigenvalues of 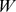) will provide a good approximation of the basis vectors 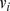 of the HO GSVD subspace (Figure S4).

### Cluster nodes selection and cluster validation

#### Cluster nodes selection

After we identified 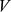 using our approximate HO GSVD, the input datasets can be reordered by using the informative vectors of 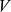, so that nodes that share similar characteristics tend to cluster into the same diagonal block of the co-expression matrix 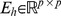 or in the same block formed by reordered rows of the expression matrix 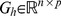. For each selected 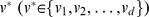, the identification of a sub-set of nodes that have significantly large similarity with each other as compared with the rest of the nodes is obtained using a Gaussian Mixture Model (GMM). Similarly to [64], here we assume that each informative 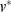 can be decomposed into two components since we are interested in learning how likely the distribution of 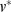 is unimodal (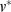 cannot be used for data clustering) or bimodal. Moreover, we assume that the two components (groups) are not treated symmetrically since the component with smaller weight identifies the cluster of nodes with high similarity. Conditionally on 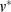, the posterior probability that the 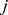
th node belongs to 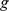
th component, 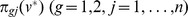 is calculated using the function fdrtool in the *R* package *fdrtool* [65] with the normal mixture distribution option. Nodes are classified into the two components depending upon the (local) misclassification error rate (MER)
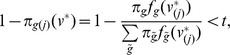
where 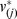 is the 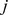
th ordered element of 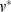, 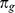 and 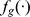 are the weight and the 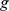
th component with smaller weight, respectively. In contrast with alternative commonly used methods [13], [16], [17], our approach does not use arbitrary parameters external to the data (apart from the MER level), such as the size of the cluster or the cluster density, to select the significant nodes.

#### Cluster validation

The C3D method integrates an automatic permutation-based approach to assess the significance of clusters across multiple conditions (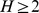). This allows to (i) identify the specific conditions where each cluster is detected and (ii) assess an empirical measure of significance for each cluster. This cluster “validation” approach can be divided into 2 steps. The first step is implemented to identify the subset of the input data 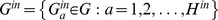 with 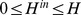, which represents the conditions where the clusters are present. Likewise, the subset 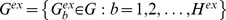 with 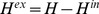 indicates the conditions where the cluster is not present. We used an estimate of the cluster “quality” 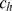 (see below) to calculate an *individual P-value* (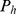) indicating the significance of one candidate cluster in each dataset 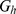. For each dataset 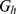 separately, 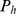 is computed as the proportion of the cluster quality calculated from random samples that exceed 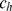, where 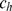 indicates the *individual cluster quality* in 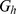. In the second step, we evaluate the overall significance (*overall P-value* or 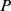) of the cluster present in conditions 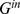 but not in 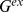. The *overall P-value* for the target cluster is computed as the proportion of cluster quality of the random samples that exceed 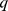, where 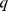 represents the *overall cluster quality* in all input datasets. In both steps, we used incremental permutations to generate random samples in a computationally efficient way and regard a *P*-value (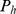 and 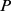) below 0.05 as significant.

The cluster “quality” measurements (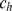 and 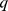) are defined as follows:
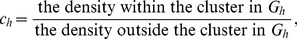
(8)

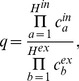
(9)where 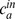 represents the cluster quality 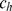 calculated in the condition 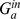 whereas 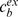 denotes 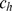 calculated in 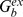. The cluster density for the weighted graphs was calculated as previously shown [14]. More details are provided and discussed in Text S1.

### Experimental data description

We selected two large gene expression datasets from rats and humans, where genome-wide expression profiles were assessed in the same subject/animal across multiple tissues. The rat datasets consisted of microarray-based expression profiles for 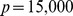 probe sets that were measured in adrenal, aorta, fat, kidney, left ventricle, liver and skeletal muscle tissues in a panel of 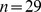 recombinant inbred rat strains [29]. Microarray expression data were retrieved from ArrayExpress, http://www.ebi.ac.uk/arrayexpress/, (skeletal muscle, E-TABM-458; aorta, E-MTAB-322; liver, E-MTAB-323, fat and kidney, E-AFMX-7; heart, MIMR-222; adrenal, E-TABM-457); gene expression summaries were derived using robust multichip average (RMA) algorithm [66] and normalized using Z-score transformation before analysis with C3D. The human data were retrieved from the Gene Expression Omnibus (GEO) database (www.ncbi.nlm.nih.gov/geo) under accession number GSE38805. Briefly, total RNA from the VZ, ISVZ, OSVZ, and CP of six 13–16 wk postconception human fetuses was isolated from laser-capture microdissected Nissl-stained cryosections of dorsolateral telencephalon (see [32] for additional details on experimental procedures). RNA-seq data were expressed as fragments per kilobase of exon per million fragments mapped (FPKM) values and normalized on log2 scale, yielding an expression matrix of 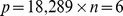 in 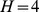 neocortex regions, which were analyzed by C3D.

### Software availability

The Matlab implementation of the C3D algorithm, detailed instructions to run the code and an example of the simulated datasets used in these studies can be downloaded from http://www.csc.mrc.ac.uk/Research/Groups/IB/IntegrativeGenomicsMedicine/ contact information: enrico.petrettocsc.mrc.ac.uk or xiaolin.xiaocsc.mrc.ac.uk

## Supporting Information

Figure S1Comparison between C3D, WGCNA and DiffCoEx methods for analysis of simulated datasets consisting of 5,000 genes and 10 observations in 7 conditions. SD, standard deviation measured over 20 replicated datasets; dashed line, 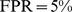.(TIFF)

Figure S2
*Top*, computational time required by C3D algorithm to analyze 1,000 genes in 25 conditions (top left) and 10,000 genes in 3 conditions (top right). *Bottom*, comparison of computational times of C3D, WGCNA and DiffCoEx methods for analysis of 1,000 (left) and 10,000 (right) genes in 7 conditions. All comparison were carried out using a standard desktop computer (Mac Pro, 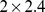 GHz Quad-core Intel Xeon with 20 Gb RAM).(TIFF)

Figure S3We assessed whether *rat cluster 1* genes were significantly co-expressed in human heart and liver tissues. We carried out genome-wide co-expression network analysis by Graphical Gaussian models using human gene expression datasets from the heart (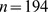 subjects, GEO: GSE5406) and liver tissue (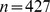 subjects, GEO: GSE9588). We first selected the top 10,000 varying genes in each dataset using co-variance filtering and then calculated the partial correlation matrix. We then tested whether the human-rat orthologous genes of rat cluster 1 (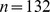 annotated genes) had significant partial-correlations more than what expected in 10,000 randomly sampled networks. Out of 132 genes in *rat cluster 1*, 132 and 115 had human-rat orthologous genes in heart and liver expression datasets, and included all Hsp and cardiomyopathy genes identified in the rat (except for PLEC which was not present in the human liver dataset). At 5% FDR we detected 95 genes (forming 194 significant edges) in the heart and 108 genes (forming 439 significant edges) in the liver tissue, respectively. We report the density of the number of edges observed in 10,000 randomly sampled networks and number of significant edges detected in each tissue (indicated by the red dot). The dashed red line indicates the 95 percentile of the distribution. For each tissue, the *P*-values were calculated as follows: 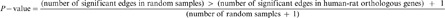

(TIFF)

Figure S4Correlation between the solutions of the approximate HO GSVD (eigenvectors of 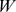) and simulated cluster structures for different noise levels (i.e., proportion of the error variance, ranging from 20% to 80%). For each dataset, we simulated 1,000 genes and 3 independent cluster structures: one “common” cluster structure is present simultaneously in 3 conditions (*left panels*), one “differential” cluster structure is present in 2 conditions (*middle panels*) and another “differential” cluster structure is present in 1 condition (*right panels*). For each level of error variance (x-axes), 100 independent replicates were generated and the absolute correlations between the first three eigenvectors of 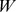 and the simulated patterns are reported as median and interquartile range (y-axes). The quality of the pattern reconstruction decreases when the error variance increases for all cluster structures. As expected, the drop is higher for the cluster structure that is unique to one condition since it explains a lower amount of the total variance across the three conditions. Please refer to Text S1 for additional details on the simulated data.(TIFF)

Table S1 Co-expression clusters identified by C3D in the rat.
(XLSX)

Table S2 Functional annotation of co-expression clusters identified in rat.
(XLSX)

Table S3 Disease Enrichment for rat cluster 1. R: Ratio of enrichment for disease associated genes, rawP: enrichment *p*-value from hypergeometric test, adjP: enrichment *p*-value adjusted for the multiple testing.
(XLSX)

Table S4 Co-expression clusters identified by C3D in human fetal neocortex.
(XLSX)

Table S5 Functional annotation of co-expression clusters identified in human fetal neocortex.
(XLSX)

Text S1 Supporting methods.
(PDF)
