# Supplementary figures and images for: Multi-tissue Analysis of Co-expression Networks by Higher-Order Generalized Singular Value Decomposition Identifies Functionally Coherent Transcriptional Modules
Source: PLoS Genet. 2014 Jan 2;10(1):e1004006. doi: 10.1371/journal.pgen.1004006 (PMC3879165; doi:10.1371/journal.pgen.1004006)

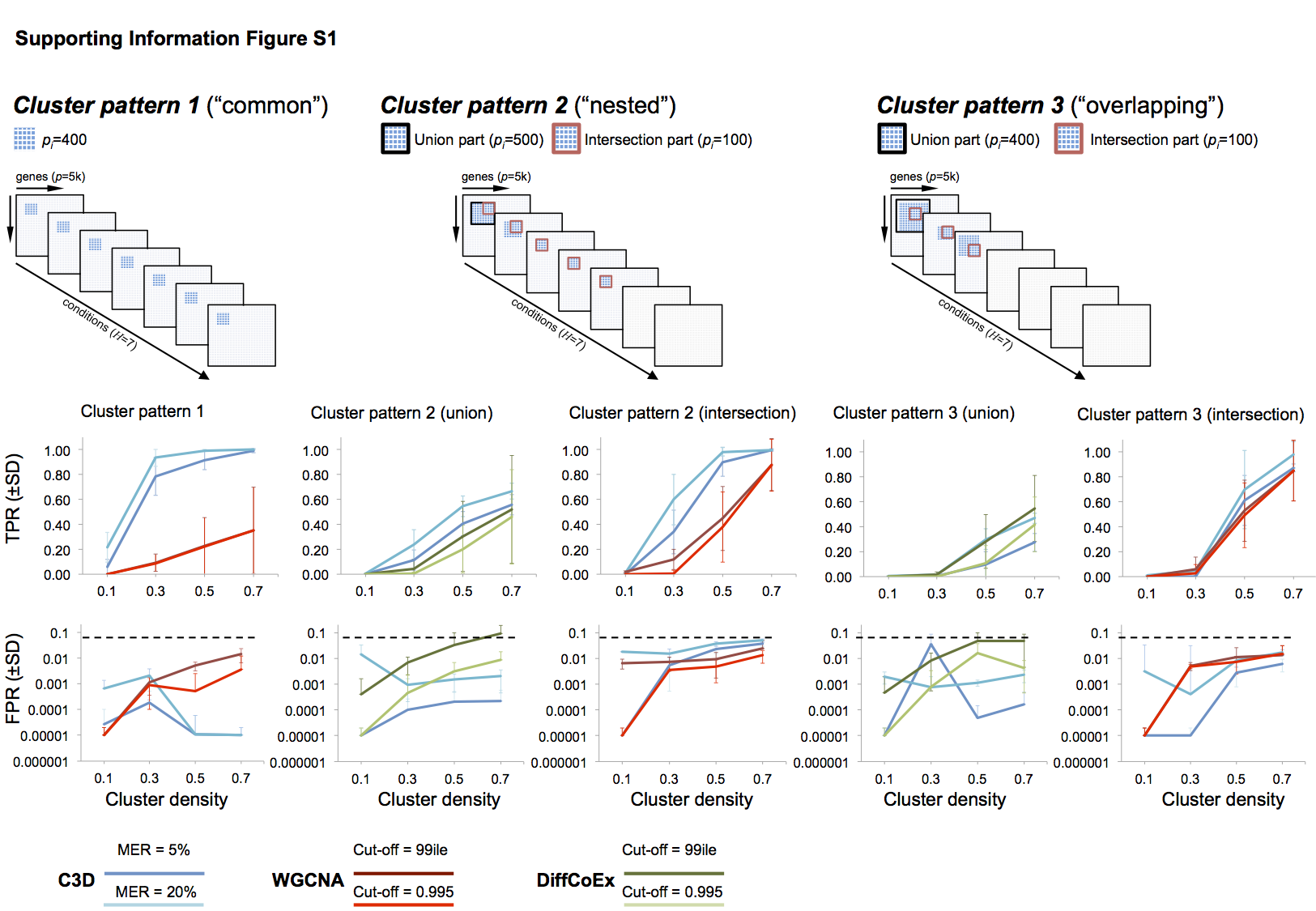

Supplement: Figure S1 — Comparison between C3D, WGCNA and DiffCoEx methods for analysis of simulated datasets consisting of 5,000 genes and 10 observations in 7 conditions. SD, standard deviation measured over 20 replicated datasets; dashed line, . (TIFF) [file pgen.1004006.s001.tiff]

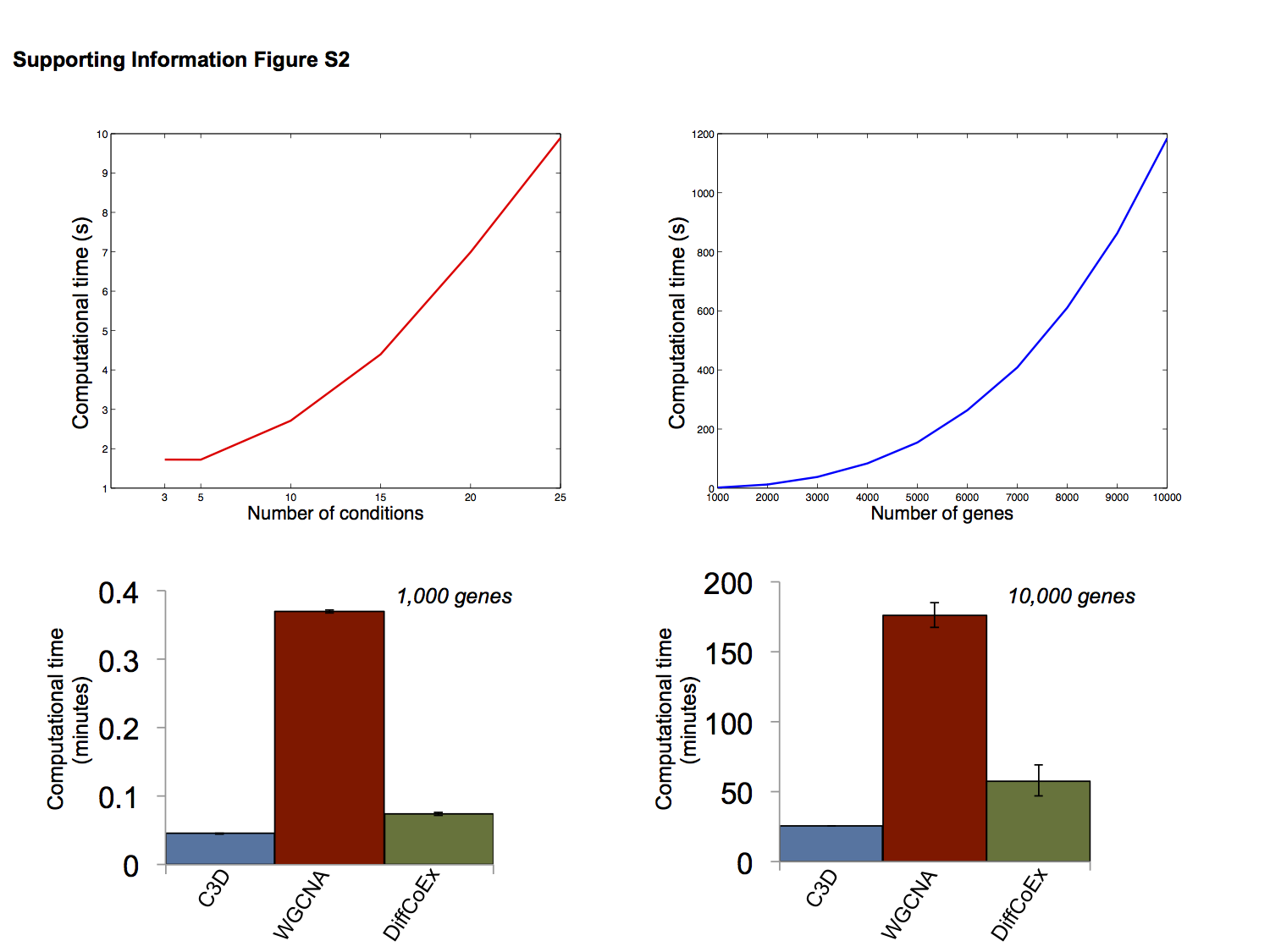

Supplement: Figure S2 — Top, computational time required by C3D algorithm to analyze 1,000 genes in 25 conditions (top left) and 10,000 genes in 3 conditions (top right). Bottom, comparison of computational times of C3D, WGCNA and DiffCoEx methods for analysis of 1,000 (left) and 10,000 (right) genes in 7 conditions. All comparison were carried out using a standard desktop computer (Mac Pro, GHz Quad-core Intel Xeon with 20 Gb RAM). (TIFF) [file pgen.1004006.s002.tiff]

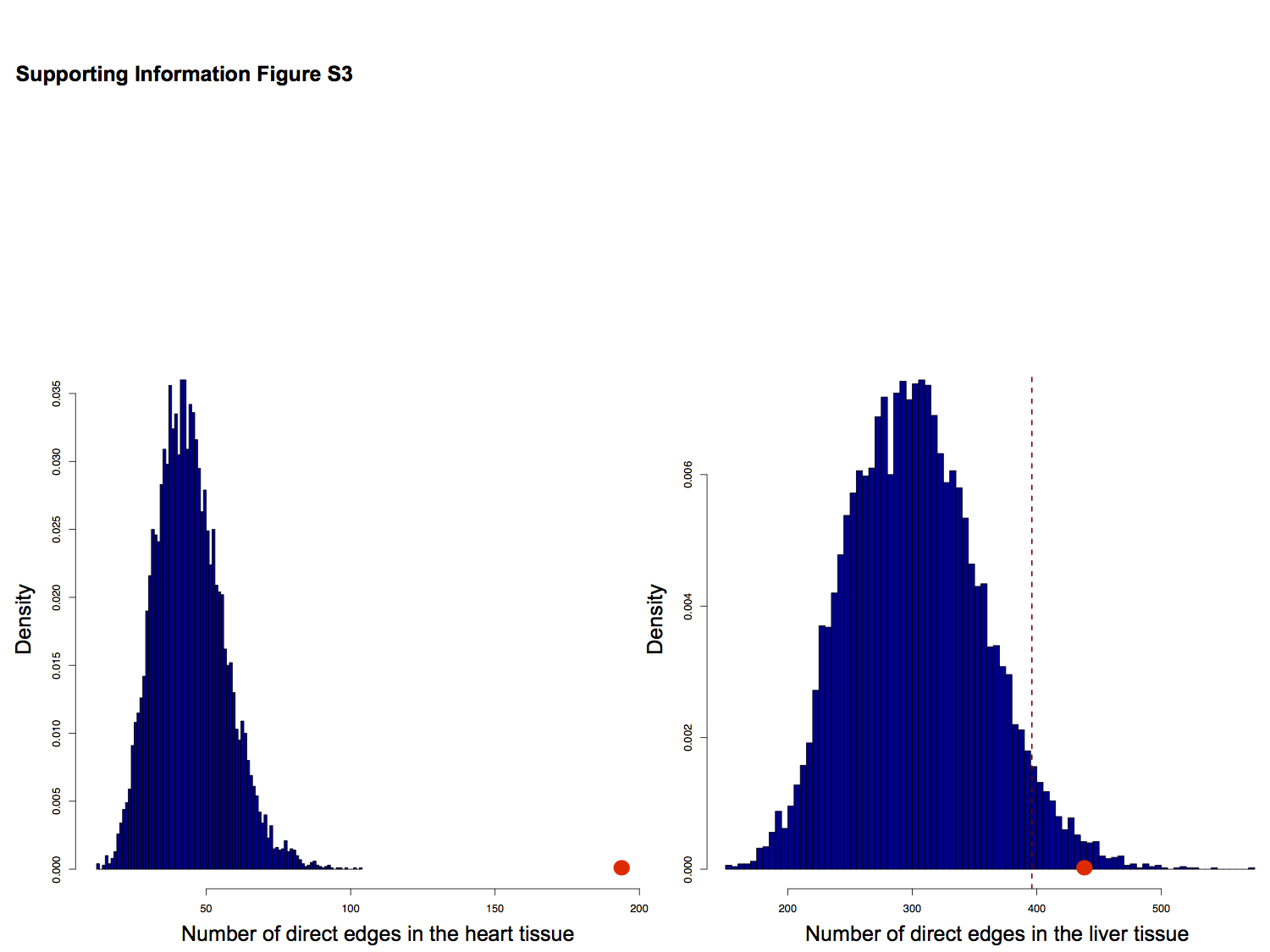

Supplement: Figure S3 — We assessed whether rat cluster 1 genes were significantly co-expressed in human heart and liver tissues. We carried out genome-wide co-expression network analysis by Graphical Gaussian models using human gene expression datasets from the heart ( subjects, GEO: GSE5406) and liver tissue ( subjects, GEO: GSE9588). We first selected the top 10,000 varying genes in each dataset using co-variance filtering and then calculated the partial correlation matrix. We then tested whether the human-rat orthologous genes of rat cluster 1 ( annotated genes) had significant partial-correlations more than what expected in 10,000 randomly sampled networks. Out of 132 genes in rat cluster 1, 132 and 115 had human-rat orthologous genes in heart and liver expression datasets, and included all Hsp and cardiomyopathy genes identified in the rat (except for PLEC which was not present in the human liver dataset). At 5% FDR we detected 95 genes (forming 194 significant edges) in the heart and 108 genes (forming 439 significant edges) in the liver tissue, respectively. We report the density of the number of edges observed in 10,000 randomly sampled networks and number of significant edges detected in each tissue (indicated by the red dot). The dashed red line indicates the 95 percentile of the distribution. For each tissue, the P-values were calculated as follows: (TIFF) [file pgen.1004006.s003.tiff]

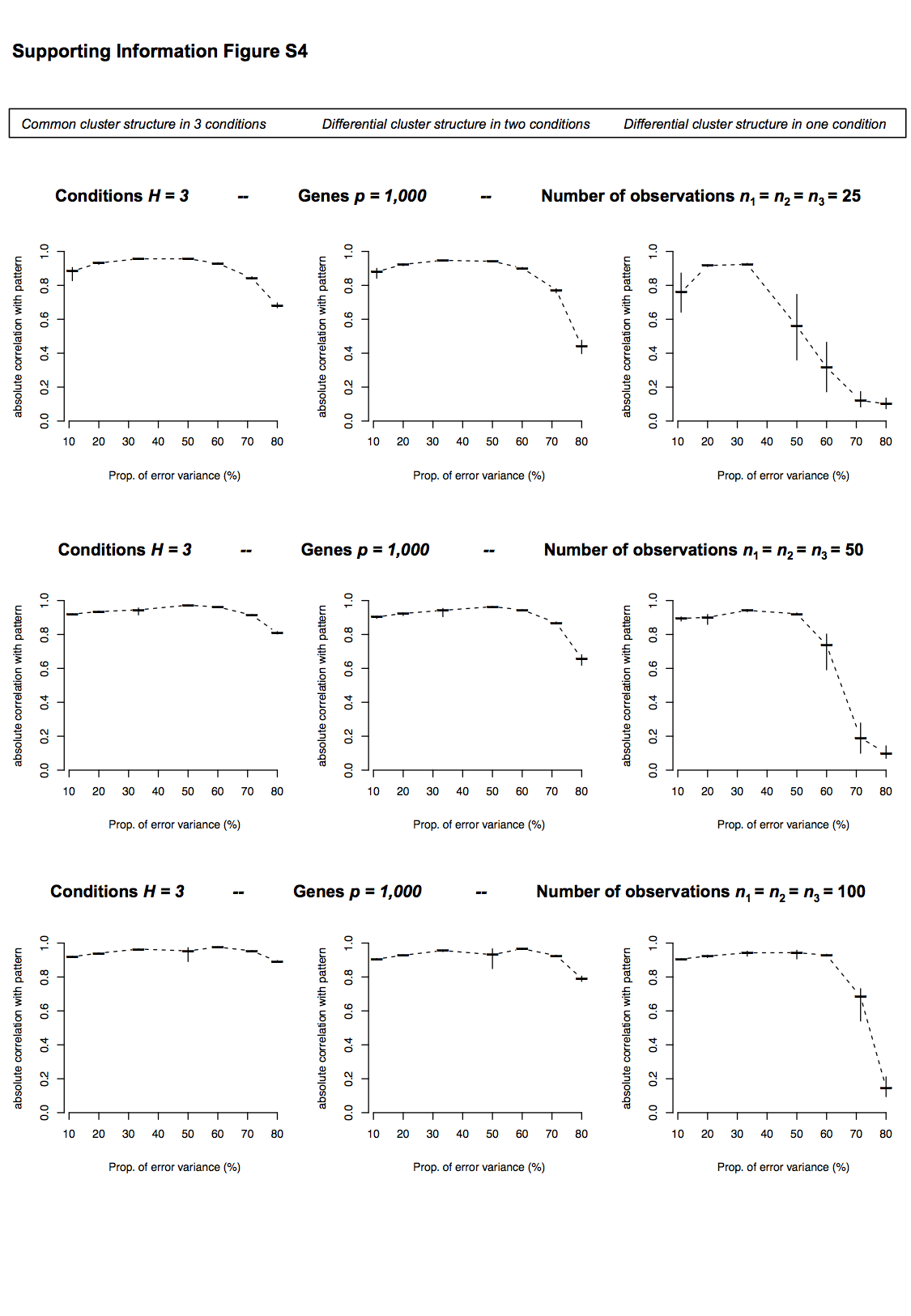

Supplement: Figure S4 — Correlation between the solutions of the approximate HO GSVD (eigenvectors of ) and simulated cluster structures for different noise levels (i.e., proportion of the error variance, ranging from 20% to 80%). For each dataset, we simulated 1,000 genes and 3 independent cluster structures: one “common” cluster structure is present simultaneously in 3 conditions (left panels), one “differential” cluster structure is present in 2 conditions (middle panels) and another “differential” cluster structure is present in 1 condition (right panels). For each level of error variance (x-axes), 100 independent replicates were generated and the absolute correlations between the first three eigenvectors of and the simulated patterns are reported as median and interquartile range (y-axes). The quality of the pattern reconstruction decreases when the error variance increases for all cluster structures. As expected, the drop is higher for the cluster structure that is unique to one condition since it explains a lower amount of the total variance across the three conditions. Please refer to Text S1 for additional details on the simulated data. (TIFF) [file pgen.1004006.s004.tiff]
