# Supplementary material for: Multi-tissue Analysis of Co-expression Networks by Higher-Order Generalized Singular Value Decomposition Identifies Functionally Coherent Transcriptional Modules
Source: PLoS Genet. 2014 Jan 2;10(1):e1004006. doi: 10.1371/journal.pgen.1004006 (PMC3879165; doi:10.1371/journal.pgen.1004006)
Supplement: Text S1 — Supporting methods. (PDF) [file pgen.1004006.s010.pdf]

Multi-tissue analysis of co-expression networks by Higher-Order Generalized Singular Value Decomposition identifies functionally coherent transcriptional modules

SUPPORTING INFORMATION

Text S1. Supporting Methods

Xiaolin Xiao[1], Aida Moreno-Moral[1], Maxime Rotival[1], Leonardo Bottolo[2] and Enrico Petretto[1*]

1. Medical Research Council (MRC) Clinical Sciences Centre, Faculty of Medicine, Imperial College, London W12 0NN, UK.
2. Department of Mathematics, Imperial College, London SW7 2AZ, UK.
∗ Corresponding author: enrico.petretto@csc.mrc.ac.uk.

Xiaolin Xiao (xiaolin.xiao@csc.mrc.ac.uk)
Aida Moreno Moral (aida.moreno-moral11@imperial.ac.uk)
Maxime Rotival (m.rotival@imperial.ac.uk)
Leonardo Bottolo (l.bottolo@imperial.ac.uk)
Enrico Petretto (enrico.petretto@csc.mrc.ac.uk)

Contents

S1 The approximate HO-GSVD in the full rank case **3**

S1.1 Analysis of 2 full rank co-expression matrices 3

S1.2 Analysis of more than 2 full rank co-expression matrices 4

S2 Cluster nodes selection and empirical validation **5**

S2.1 Empirical cluster validation 5

S2.1.1 Cluster quality measures 7

S3 Details on simulated datasets **9**

S3.1 Identification of common and differential structures in the non full rank case . . 9

S3.2 Comparison with WGCNA and DiffCoEx for Cluster Identification 9

S4 Annotation of gene co-expression networks **10**

S5 Details on parameterisation of WGCNA and DiffCoEx **11**

S1 The approximate HO-GSVD in the full rank case

One of the interesting properties of our approximate HO-GSVD, is its ability to revert to the standard GSVD (if $H = 2$) and the HO GSVD (if $H > 2$) in the special case where the input matrices G_h are full column rank, as detailed below.

S1.1 Analysis of 2 full rank co-expression matrices

Our algorithm searches for a common decomposition of a set of matrices $G_h \in \mathbb{R}^{n\times p}$ by finding the eigenvector decomposition of $W \in \mathbb{R}^{p\times p}$ defined as

$$W = \frac{1}{H(H-1)} \sum_{h=1}^{H} \sum_{r>h}^{H} (E_h E_r^+ + E_r E_h^+), \tag{1}$$

with $E_h = G_h^T G_h \in \mathbb{R}^{p\times p}$ representing a symmetric positive co-expression matrix for condition H, and with E^+. denoting the Moore-Penrose inverse of the matrix E.

In the case for $H = 2$, this expression of W reduces to

$$W = \frac{1}{2}\left(E_1 E_2^+ + E_2 E_1^+\right) \tag{2}$$

which can be rewritten as follows when the matrices G_h are full column rank

$$W = \frac{1}{2}\left(E_1 E_2^{-1} + E_2 E_1^{-1}\right) \tag{3}$$

Link with the Generalised Singular Value Decomposition (GSVD)

Under this scenario, the Generalised Singular Value Decomposition (GSVD) for a pair of full column rank matrices matrices $G_1 \in \mathbb{R}^{l\times n}$ with $l \geq n$ and $G_2 \in \mathbb{R}^{m\times n}$ is given by

$$G_1 = U_1 \Sigma_1 X^{-1} \qquad \text{and} \qquad G_2 = U_2 \Sigma_2 X^{-1}, \tag{4}$$

where $U_1 \in \mathbb{R}^{l\times l}$ and $U_2 \in \mathbb{R}^{m\times m}$ are both orthogonal, $\Sigma_1 \in \mathbb{R}^{l\times n}$ and $\Sigma_2 \in \mathbb{R}^{m\times n}$ are diagonal $\Sigma_1 = \text{diag}(\sigma_{1,1}, \sigma_{1,2}, \ldots, \sigma_{1,n})$ and $\Sigma_2 = \text{diag}(\sigma_{2,1}, \sigma_{2,2}, \ldots, \sigma_{2,q})$ with non-negative entries, $q = \min(m, n)$ and $X \in \mathbb{R}^{n\times n}$ is invertible [1]. In this case, it can be shown that $\Sigma_1^T \Sigma_1 + \Sigma_2^T \Sigma_2 = I_n$ [1] and by a simple reordering of the rows of X^{-1}, the diagonal entries in

Σ_1 can be chosen to be with increasing order $0 \leq \sigma_{1,1} \leq \sigma_{1,2} \leq \cdots \leq \sigma_{1,n}$ while those of Σ_2 are in decreasing order $\sigma_{2,1} \geq \sigma_{2,2} \geq \cdots \geq \sigma_{2,q} \geq 0$. The ratios $\sigma_{1,i}/\sigma_{2,i}$ are the *generalised singular values* of G_1 and G_2. The rows of X^{-1} provide a common basis for the decomposition of G_1 and G_2. Therefore, by defining $V = (X^{-1})^T$ we can rewrite the GSVD decomposition as

$$G_1 = U_1 \Sigma_1 V^T \qquad \text{and} \qquad G_2 = U_2 \Sigma_1 V^T, \tag{5}$$

where $U_1 \in \mathbb{R}^{l \times l}$ and $U_2 \in \mathbb{R}^{m \times m}$ are orthogonal, $V \in \mathbb{R}^{n \times n}$ is nonsingular. In practical applications, this GSVD of $G_1 \in \mathbb{R}^{l \times n}$ and $G_2 \in \mathbb{R}^{m \times n}$ can easily be obtained using the Paige's algorithm [2].

Therefore in the full rank case ($H = 2$), the existence of an exact decomposition of $G_h = U_h \Sigma_h V^T$ ($h = 1, 2$) with $U_h \in \mathbb{R}^{p \times p}$ orthogonal is guaranteed, and we can then rewrite E_1 and E_2 as follows :

$$E_1 = G_1^T G_1 = V \Sigma_1^2 V^T$$

and

$$E_2 = G_2^T G_2 = V \Sigma_2^2 V^T.$$

Plugging E_1 and E_2 into (3), we have the following decomposition of $W \in \mathbb{R}^{p \times p}$

$$\begin{aligned} W &= \frac{1}{2}(E_1 E_2^{-1} + E_2 E_1^{-1}) \\ &= \frac{1}{2} V (\Sigma_1^2 \Sigma_2^{-2} + \Sigma_2^2 \Sigma_1^{-2}) V^{-1} \end{aligned} \tag{6}$$

where the diagonal entries of $\Sigma_h^2 (h = 1, 2)$ are the square of those in Σ_h and $V \in \mathbb{R}^{p \times p}$ is invertible. Equation (6) can be interpreted as the eigen-decomposition of W, where the columns of V (i.e., v_k with $k = 1, 2, \ldots, p$) are the eigenvectors and the corresponding eigenvalues are the diagonal entries of the matrix $\Sigma_1^2 \Sigma_2^{-2} + \Sigma_2^2 \Sigma_1^{-2}$. Hence the eigenvalue decomposition of W as defined in (1) coincides with the standard GSVD in the case $H = 2$, with G_h full column rank.

S1.2 Analysis of more than 2 full rank co-expression matrices

It is straightforward to see that in the full rank case our algorithm reverses to the HO-GSVD defined by Ponnapali [3]. Indeed, in this case, The Moore-Penrose inverse of E_h, E_h^+ is equal to the standard inverse of E_h and W can be rewritten as

$$W = \frac{1}{H(H-1)} \sum_{h=1}^{H} \sum_{r>h}^{H} (E_h E_r^{-1} + E_r E_h^{-1}), \tag{7}$$

which is the formulation of W used in [3]. Therefore in the case were $p \leq n$, the proposed algorithm is equivalent to using the HO GSVD proposed by Ponnapali et al., with the only difference that the G_h matrices in our our case are transposed compared to those used in the framework proposed in [3].

S2 Cluster nodes selection and empirical validation

S2.1 Empirical cluster validation

We developed an automatic computational procedure for "validation" of candidate clusters and determine their statistical significance across conditions. A similar "validation" procedure was introduced by [4], which was based on the calculation of a quality measure for the cluster (i.e., c_h, the density calculated inside the cluster over the density calculated outside the cluster in condition h, see S2.1.1) and on the empirical P-values by permutations. However, the cluster quality measure and empirical P-values were designed only to analyse two conditions and assess the significance of the "differential" clusters (i.e., clusters present in one but not in the other condition). Here, we have extended this approach to the general case of multiple input datasets G_h ($h = 1, \dots H$ and $H \geq 2$) and to assess the significance of both "common" and "differential" clusters.

Suppose that we can reorder all input data (G_h, $h = 1, \dots, H$ and $H \geq 2$) using an informative column vector v^* of V such that we can identify a group of τ nodes forming a candidate cluster $s^\diamond$ present in H^{in} conditions ($G_a^{in} \in G$, $a = 1, \dots, H^{in}$, $0 \leq H^{in} \leq H$) but not in the other H^{ex} conditions ($G_b^{ex} \in G$, $b = 1, \dots, H^{ex}$ and $H^{ex} = H - H^{in}$). Then we can use the following two-step procedure to assess the significance of the candidate cluster and calculate the empirical P-value as follows:

Step 1. Apply the random permutation test separately to each input dataset G_h and identify the conditions G^{in} containing $s^\diamond$, as follows:

- **Step 1.1.** compute the cluster quality measure c_h (see (8) in S2.1.1) for the cluster $s^\diamond$ consisting of τ nodes in each reordered dataset ${G_h}^*$ (G_h reordered by column v^*);
- **Step 1.2.** randomise the nodes within each dataset and compute the cluster quality $\hat{c}_h$ for a set of τ nodes separately in each randomised dataset $\hat{G}_h$; repeat this step M times;
- **Step 1.3.** separately for each dataset G_h compute the P-value (P_h) as the number of proportion of $\hat{c}_h$ samples that exceed c_h in M permutations;

Step 1.4. we will use P_h to identify the datasets (G^{in}) where the candidate cluster $s^\diamond$ is present: for each dataset if the P-value is lower than a given threshold (defined by the user, default value 0.05), we will consider the cluster $s^\diamond$ present in the corresponding dataset G_h and so we will put G_h into the set G^{in}, otherwise we will put the dataset G_h into the set G^{ex}. The sets G^{in} and G^{ex} are passed as input for Step 2 and will used to calculate the overall cluster quality measure q across multiple conditions.

Step 2. Apply the random permutation test to all datasets and determine the overall significance of the candidate cluster $s^\diamond$ of τ nodes which is present in G^{in} but not in G^{ex}. This step will generate an single P-value (P) for the overall significance of the cluster as follows:

Step 2.1. compute an overall cluster quality q across multiple conditions (see (9) in S2.1.1) for the candidate cluster $s^\diamond$ consisting of τ nodes, which is present in G^{in} but not in G^{ex};

Step 2.2. randomise the nodes within each condition and compute the cluster quality $\hat{q}$ for a set of τ nodes; repeat this M times;

Step 2.3. compute the final P-value (P) for the cluster $s^\diamond$ as the proportion of $\hat{q}$ samples that exceed q in M permutations.

In summary, this two-step procedure generates two empirical P-values: (Step 1) P_h for cluster $s^\diamond$ in each condition h and (Step 2) P for for cluster $s^\diamond$ in all conditions where the cluster is present (G^{in}). For convenience, we refer to P_h as the *individual P-value* and P as the *overall P-value*. We use the *individual P-value* in Step 1 to identify the conditions G^{in} where the candidate cluster $s^\diamond$ is detected and in Step 2 we calculate the *overall P-value* to assess the overall significance of the cluster in conditions G^{in}.

While previous studies showed that $M = 1000$ randomisations are usually sufficient to estimate the cluster significance [4], more randomisations are typically suggested to assess the significance of larger and more complex cluster structures. Since large number of permutations (e.g., $M \geq 10,000$) would be computational expensive in the case of large datasets with several thousands of features measured across many conditions, in our algorithm we have implemented incremental permutations in both Step 1.2 and Step 2.2. For example, in Step 1, we randomise each condition for a small number of times; then we compute P_h for each dataset and we increase the number of permutations only for those G_h whose corresponding P_h is below a given threshold P^* (i.e., $P_h < P^*$). For the G_h with $P_h \geq P^*$, we will stop the permutation procedure and assign the G_h to the set of conditions where the cluster in not present (G^{ex}). A similar

procedure based on incremental permutations is employed in Step 2. The user can specify the minimum number of permutations (default 100), maximum number of permutations (default $1,000$) and the critical P^* used to stop incremental permutations (default $P^* = 0.05$).

S2.1.1 Cluster quality measures

The permutation based procedure uses two cluster quality measures: the *individual cluster quality* c_h and the *overall cluster quality* q. The *individual cluster quality* c_h for cluster $s^\diamond$ is defined on each dataset G_h as

$$c_h = \frac{\text{the density within the cluster in } G_h}{\text{the density outside the cluster in } G_h} \tag{8}$$

where the density $f(s^\diamond)$ corresponds to the average weight calculated for a group of nodes, as previously described [4]. The *overall cluster quality* q is used to assess the relative cluster density for both "differential" and "common" clusters and it is calculated across all datasets as

$$q = \frac{\prod_{a=1}^{H^{in}} c_a^{in}}{\prod_{b=1}^{H^{ex}} c_b^{ex}}, \tag{9}$$

where c_a^{in} represents the cluster quality c_h calculated in condition G_a^{in} where the candidate cluster $s^\diamond$ was detected, whereas the c_b^{ex} denotes the c_h computed from the other condition G_b^{ex} where the cluster $s^\diamond$ was not detected. When $s^\diamond$ is a "common" cluster detected in all conditions (i.e., $G^{in} = G$ and $H^{in} = H$), (9) is equivalent to $q = \prod_{h=1}^{H} c_h$.

Table SN1 summarises cluster quality measures (q) used in the case of "common" and "differential" clusters selected by the HO-GSVD-based algorithm and by the GSVD-based algorithm.

	GSVD	HO-GSVD
Cluster quality measure for "differential" cluster	$q = \frac{c_1}{c_2}$	$q = \frac{\prod_{a=1}^{H^{in}} c_a^{in}}{\prod_{b=1}^{H^{ex}} c_b^{ex}}$
Cluster quality measure for "common" cluster	$q = c_1 \times c_2$	$q = \prod_{h=1}^{H} c_h$

Table SN1: Comparison of cluster quality measures used to "validate" clusters obtained by the GSVD-based algorithm and the HO-GSVD-based algorithm.

Alternative cluster quality measures

In addition to the above mentioned c_h and q, we have designed different cluster quality measures which are implemented in our algorithm and can be chosen by the user:

Individual cluster quality measure:

$$c*_h = \text{the density within the cluster in } G_h$$

is a simpler quality measure than the c_h defined in (8) which is less suitable for comparing several conditions (≥ 2) where the background levels show large variations across the G_h.

Overall cluster quality measures: Alternative formulations of q that are implemented in our algorithm are summarised as follows :

Sum based	$q = \frac{\sum_{a=1}^{H^{in}} c_a^{in}}{\sum_{b=1}^{H^{ex}} c_b^{ex}}$
Arithmetic mean based	$q = \frac{(\sum_{a=1}^{H^{in}} c_a^{in})/H^{in}}{(\sum_{b=1}^{H^{ex}} c_b^{ex})/H^{ex}}$
Geometric mean based	$q = \frac{(\prod_{a=1}^{H^{in}} c_a^{in})^{\frac{1}{H^{in}}}}{(\prod_{b=1}^{H^{ex}} c_b^{ex})^{\frac{1}{H^{ex}}}}$
Product based	$q = \frac{H^{in}(H^{in}-1)\prod_{a=1}^{H^{in}} c_a^{in}}{H^{ex}(H^{ex}-1)\prod_{b=1}^{H^{ex}} c_b^{ex}}$
Power based	$q = \frac{\sum_{a=1}^{H^{in}}\sum_{d>a}^{H^{in}} c_a^{in}(c_d^{in})^{-1}}{\sum_{b=1}^{H^{ex}}\sum_{e>b}^{H^{ex}} c_b^{ex}(c_e^{ex})^{-1}}$
Mixture based	$q = \frac{(\sum_{a=1}^{H^{in}} c_a^{in})/(\sum_{a=1}^{H^{in}}\sum_{d>a}^{H^{in}} c_a^{in}(c_d^{in})^{-1})}{(\sum_{b=1}^{H^{ex}} c_b^{ex})/(\sum_{b=1}^{H^{ex}}\sum_{e>b}^{H^{ex}} c_b^{ex}(c_e^{ex})^{-1})}$

Table SN2: Alternative formulations for overall cluster quality measure q.

When $s^\diamond$ is a "common" cluster detected in all conditions (i.e., $G^{in} = G$ and $H^{in} = H$) we set the denominator of the above defined cluster quality measure q to 1. From our simulation studies (see main text) we empirically observed that all the above clusters quality measures q work equally well to detect "common" clusters present in all conditions. However, to detect "differential" clusters the product based quality measure was more efficient than (9) and the arithmetic mean based quality measure performed better than other quality measures when the cluster is present in all conditions but one (data not shown).

S3 Details on simulated datasets

S3.1 Identification of common and differential structures in the non full rank case

To test the ability of the modified HO-GSVD algorithm to capture the hidden covariates in presence of a "noisy" HO-GSVD decomposition, we simulated 100 datasets composed of either 200 or 1000 genes observed under 3 conditions with 25 or 50 samples per condition. In each dataset, we first simulated and exact HO-GSVD decomposition of the form $G_h = U_h \Sigma_h V^T$ $(h = 1, 2, \ldots, H)$ before adding a random gaussian noise with variance constant variance σ^2 to each of the G_h matrices.

To simulate the initial decomposition, we first simulated three independent gaussian patterns v_i respectively present in one, two, or three conditions. We then simulated gaussian orthonormal left basis vectors U_h such that we have the exact covariance structure $G_h^T G_h = V \Sigma_h^2 V^T$ with Σ_h a diagonal matrix with the elements σ_h, i such that

$$\sigma_{h,1} = 1, \text{ for } h = 1, 2, 3$$

$$\sigma_{h,2} = 1 \text{ if } h = 1, 2, \text{ 0 otherwise}$$

$$\sigma_{h,3} = 1 \text{ if } h = 3 \text{ 0 otherwise}$$

Finally, we added a gaussian independent noise $\epsilon\ N(0, \sigma^2)$ to the data to test the robustness of the method to the presence of noise. The simulations were carried out for various values of σ in order to test the effect of increasing noise on the quality of the resulting decomposition. In our simulations, we define the noise as the total share of variance explained by the noise in the dataset.

$$\text{Prop of Error Variance} = \frac{\sigma^2}{\sum_k \sigma_{h,k}^2 + \sigma^2} = \frac{\sigma^2}{2 + \sigma^2}$$

S3.2 Comparison with WGCNA and DiffCoEx for Cluster Identification

We simulated 20 replicates of 4 groups of synthetic datasets. Each of these datasets includes: $H = 7$ conditions, $p = 5,000$ nodes, $n = 30$ observations and 3 different clusters. Each group of datasets corresponds to a given cluster density (see below). Three distinct clusters were generated within each dataset:

Cluster pattern 1 : "common" cluster present in all 7 conditions where the cluster size is identical across conditions;

Cluster pattern 2 : "nested" cluster present in 5 out of 7 conditions (conditions 1, 3, 4, 5 and 7) where the cluster size is incremental across conditions;

Cluster pattern 3 : "overlapping" cluster present in 3 conditions (conditions 2, 3 and 7).

The structure of the "common", "nested" and "overlapping" clusters is represented in Figure 2 and described in the main text. All simulated datasets were generated in MATLAB. To compare the performance of our method against alternative methods (i.e., WGCNA, DiffCoEx) in detecting clusters with different densities (measured as the average Pearson correlation between any pair of nodes within a cluster), we generated 4 groups of datasets, each with different values of cluster density: 0.1, 0.3, 0.5 and 0.7. To this aim, we used the MATLAB routine *mvnrnd* where the non-cluster nodes are random vectors chosen from the multivariate normal distribution with zero means and covariance matrix equal to identity matrix. On the other hand, the cluster nodes are random vectors chosen from the multivariate normal distribution with zero means and a given covariance to control the dependency between the cluster nodes such that the final cluster density matches the desired levels (i.e., 0.1, 0.3, 0.5 and 0.7). The same procedure was used to generate 4 similar groups of datasets (with cluster densities 0.1, 0.3, 0.5, 0.7) and with $p = 5,000$ nodes, $n = 10$ observations in $H = 7$ conditions. We generated a total of 1,200 datasets, i.e., 560 datasets for each case with $n = 10$ and $n = 30$ observations, respectively.

S4 Annotation of gene co-expression networks

Functional enrichment analysis was carried out to assess the biological significance of the obtained gene co-expression clusters by querying DAVID (Database for Annotation, Visualization, and Integrated Discovery) [5]. DAVID is an unified resource for the analysis and visualisation of heterogeneous sources of functional annotations such as Gene Ontology (GO) terms, cellular and KEGG (Kyoto Encyclopedia of Genes and Genomes) pathways [5]. All reported enrichments were significant at 5% FDR level.

Cell- and tissue-type enrichments were investigated using the Cten (Cell Type Enrichment) tool [6]. Cten uses a database of highly expressed cell specific (HECS) genes derived from publicly available databases of gene expression profiles from 85 different cell-types. Cten determines the significance of enrichment in a specific cell-type (or tissue) using the one-tailed Fisher exact test and the final enrichment score is expressed as $-\log_{10}$ of the Benjamini-Hochberg (BH) adjusted P-value reported on a circular graph across all cell/tissue types [6].

The PASTAA algorithm was used to identify overrepresented transcription factors binding

sites motifs in the promoter of genes within each rat and human cluster [7]. PASTAA compares the ranking of genes present on each cluster with the set of genes that were used in the microarrays/RNA-seq analyses, based on predicted transcription factors binding affinities. These binding affinities were determined by a biophysical model using the Transfac [8] vertebrate transcription factors matrices and considering a genomic region of ±500 bp around the gene transcription start site. P-values were corrected for multiple testing by applying false discovery rate correction [9] and a transcription factor was considered to be significantly overrepresented if FDR ≤ 0.05.

S5 Details on parameterisation of WGCNA and DiffCoEx

WGCNA and DiffCoEx analyses were carried out in R following [10]. Since WGCNA and DiffCoEx were originally developed for network analysis in single or pairwise conditions respectively, we adapted them for analysis of multiple conditions ($H = 7$). First, we had to select a soft thresholding value (β parameter of the power function) common to all 7 conditions for the WGCNA adjacency function. For each condition, scale-free topology model fitting plots were obtained following [10] where the coefficients of determination (R^2) was plotted against a range of β values. From the plots, the β parameter value that better fitted in all the conditions was selected and applied to the correlation matrixes to obtain an adjacency matrix that represents the co-expression network. Pearson correlation matrix was used in the simulation studies and Kendall correlations were employed in the real data analyses. The next step consisted of transforming the adjacency matrix into a node-node dissimilarity matrix, which represents the distance between each pair of nodes. This was achieved by calculating the Topological Overlap Matrix (TOM) and converting it into a dissimilarity measure. For multiple-conditions analysis, we had to select a "consensus TOM" over all conditions and select cut-off values for the hierarchical clustering dendogram and the dendogram height distribution. In our comparative analysis of C3D, WGCNA and DiffCoEx methods (reported in Figure 3) we have used the following cut-off values: 0.995 for the hierarchical clustering dendogram and the 99 percentile of the dendogram height distribution. Both these values were suggested in the WGCNA guidelines [10].

Since we observed variations in true positive/false positive rates when these two parameterization were adopted in the WGCNA and DiffCoEx analyses, here we investigate how the performance of these methods change when varying values of the percentile of clustering dendogram height distribution are considered. To this aim, we have simulated $p = 1,000$ gene expression profiles in $n = 30$ samples and across $H = 7$ conditions, and 3 independent repli-

cates were generated. We considered different types of clusters that are either detected in all conditions ("common" clusters) or are specific to a subset of conditions ("differential" clusters), Figure 2. In detail, we simulated clusters of variable sizes as follows: (i) "common cluster" $C1:$ $p = 40$ nodes, (ii) "differential" cluster $C2 : p = 50$ nodes in 5 out of 7 conditions and (iii) "differential" $C3 : p = 200$ nodes in 3 out of 7 conditions. These clusters have varying densities as follows: $C1\ density = 0.464$, $C2\ density = 0.212$ and $C3\ density = 0.180$. We run WGCNA and DiffCoEx analyses using increasing percentiles of the dendogram height distribution (1, 10, 20, 40, 60, 80, 90, 99 and 99.9, which correspond to cut-off values for the hierarchical clustering dendogram 0.9690847, 0.9848499, 0.989019467, 0.9927335, 0.995847767, 0.998072267, 0.998777633, 0.999200633, 0.999233567) and report the TPR and FPR for detection of $C1$ (solid red line, WGCNA), $C2$ (solid green line, DiffCoEx) and $C3$ (dashed green line, DiffCoEx), see figure below (Figure SN1). Both WGCNA and DiffCoEx show higher TPR for increasing percentile cut-off values, eventually reaching 100%. This trend is mirrored by increased false positives at high percentile values ($\geq 90\%$ for WGCNA and $\geq 99\%$ for DiffCoEx, respectively).

Irrespective of this trend, these data show the sensitivity of both WGCNA and DiffCoEx methods on the choice of the appropriate cut-off, which must be finely tuned to each specific case and dataset in order to achieve the best compromise between TP and FP. On the contrary, our HO-GSVD-based approach is parameter free and does not require specification of *ad-hoc* parameters that need to be "tuned" on the input data. The only user-specified parameter is a statistical threshold (i.e., MER) that is used to assign genes to each cluster and control the misclassification error at a desired level. This makes C3D a useful tool for real data exploration and analysis, since the user does not need to specify unknown parameters (required by other approaches) related to the expected number of clusters or cluster density [11] or related to the optimal height cut-off in the gene clustering tree [10, 12, 13].

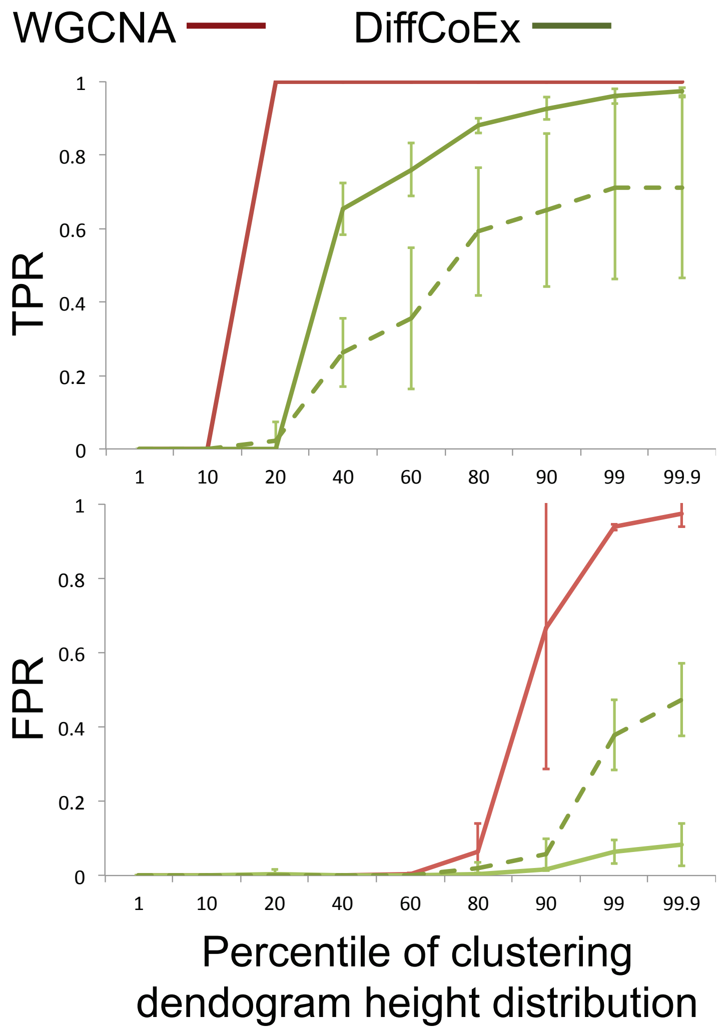

Figure SN1: Variation in TPR/FPR when different percentiles of the dendogram height distribution are used in WGCNA and DiffCoEx analyses. Solid red line, WGCNA for detection of $C1$; solid green line, DiffCoEx for detection of $C2$; dashed green line, DiffCoEx for detection of $C3$. Error bars, standard deviation measured in three replicated datasets.

References

[1] Golub GH, Van Loan CF (1996) Matrix Computations. Baltimore: Johns Hopkins University Press, third edition.

[2] Paige CC, Saunders MA (1981) Towards a generalized singular value decomposition. SIAM Journal on Numerical Analysis 18: 398-405.

[3] Ponnapalli SP, Saunders MA, Van Loan CF, Alter O (2011) A Higher-Order Generalized Singular Value Decomposition for Comparison of Global mRNA Expression from Multiple Organisms. PLoS ONE 6: e28072.

[4] Xiao X, Dawson N, MacIntyre L, Morris B, Pratt J, et al. (2011) Exploring metabolic pathway disruption in the subchronic phencyclidine model of schizophrenia with the Generalized Singular Value Decomposition. BMC Systems Biology 5: 72.

[5] Huang DW, Sherman BT, Lempicki RA (2008) Systematic and integrative analysis of large gene lists using david bioinformatics resources. Nature Protocols 4: 44-57.

[6] Shoemaker J, Lopes T, Ghosh S, Matsuoka Y, Kawaoka Y, et al. (2012) Cten: a web-based platform for identifying enriched cell types from heterogeneous microarray data. BMC Genomics 13: 460.

[7] Roider HG, Manke T, O'Keeffe S, Vingron M, Haas SA (2009) Pastaa: identifying transcription factors associated with sets of co-regulated genes. Bioinformatics 25: 435-442.

[8] Matys V, Kel-Margoulis OV, Fricke E, Liebich I, Land S, et al. (2006) TRANSFAC and its module TRANSCompel: transcriptional gene regulation in eukaryotes. Nucleic acids research 34: D108-10.

[9] Benjamini Y (1995) Controlling the false discovery rate: a practical and powerful approach to multiple testing. Journal of the Royal Statistical Society, Series B (Methodological) 57: 289-300.

[10] Zhang B, Horvath S (2005) A general framework for weighted gene co-expression network analysis. Statistical Applications in Genetics and Molecular Biology 4: Article17.

[11] Li W, Liu CC, Zhang T, Li H, Waterman MS, et al. (2011) Integrative analysis of many weighted co-expression networks using tensor computation. PLoS Comput Biol 7: e1001106.

[12] Langfelder P, Horvath S (2008) WGCNA: an R package for weighted correlation network analysis. BMC Bioinformatics 9: 559.

[13] Tesson B, Breitling R, Jansen R (2010) DiffCoEx: a simple and sensitive method to find differentially coexpressed gene modules. BMC Bioinformatics 11: 497.
